# Vaccination in Forcibly Displaced, Pediatric Populations: A Systematic Review and Meta-Analysis

**DOI:** 10.1001/jamanetworkopen.2025.16237

**Published:** 2025-06-16

**Authors:** Sargun Virk, Andrew Milewski, Nadia Khan, Celeste Cheung, Drew N. Wright, Gunisha Kaur

**Affiliations:** 1 Human Rights Impact Lab, Weill Cornell Medical College, New York, New York; 2 Department of Anesthesiology, Weill Cornell Medical College, New York, New York; 3 Weill Cornell Medical College, New York, New York; 4 Rutgers Robert Wood Johnson Medical School, New Brunswick, New Jersey; 5 Samuel J. Wood Library, C.V. Starr Biomedical Information Center, Weill Cornell Medical College, New York, New York

## Abstract

**Importance:**

Accessing forcibly displaced, pediatric populations for vaccination is challenging, and precise vaccination rates remain largely unknown.

**Objective:**

To estimate vaccination coverage and identify factors associated with vaccination in forcibly displaced, pediatric populations.

**Data Sources:**

Six databases were searched in November 2023—Ovid MEDLINE, Ovid EMBASE, PubMed, Scopus, Web of Science, and Cochrane—with no restrictions on language or publication dates. The search strategy included all appropriate controlled vocabulary and keywords for *vaccination*, *pediatrics*, and *displaced populations*.

**Study Selection:**

Included studies were original research articles investigating vaccination in pediatric populations (<19 years) that were forcibly displaced as defined by the United Nations High Commissioner for Refugees. Reviews, nonresearch articles, and qualitative studies were excluded. Screening was conducted by 2 independent reviewers with discrepancies resolved by a third reviewer. Of 1731 studies identified, 294 underwent full-text review.

**Data Extraction and Synthesis:**

Data abstraction and quality assessment followed the Preferred Reporting Items for Systematic Reviews and Meta-Analyses 2020 guidelines. A random-effects model was used to pool data. Data quality was assessed using the Downs and Black Checklist.

**Main Outcomes and Measures:**

The primary outcome was pooled vaccination coverage, defined as the proportion vaccinated. Additionally, the factors associated with vaccination were pooled from reported unadjusted odds ratios (ORs) or adjusted ORs (aORs).

**Results:**

Of the 1731 studies identified, 39 studies from 24 countries were included. Based on 11 studies, the full vaccination coverage was 21% (95% CI, 11%-36%; *I*^2^ = 98.90%). Factors associated with greater odds of vaccination included having 2 or more children in the family (OR, 1.70; 95% CI, 1.43-2.02; *I*^2^ = 0.00%; aOR, 1.81; 95% CI, 1.28-2.57; *I*^2^ = 0.00%), higher levels of guardian education (OR, 1.60; 95% CI, 1.06-2.43; *I*^2^ = 71.60%), father’s employment (OR, 2.75; 95% CI, 1.67-4.53; *I*^2^ = 0.00%), higher household income (OR = 1.33; 95% CI, 1.29-1.37; *I*^2^ = 0.00%), housing stability (OR, 2.80; 95% CI, 2.10-3.73; *I*^2^ = 0.00%; aOR, 2.62; 95% CI, 1.55-4.40; *I*^2^ = 0.00%), and residing in an area with more access to health care services (OR, 2.11; 95% CI, 1.44-3.09; *I*^2^ = 0.00%), while being forcibly displaced was associated with lower odds of vaccination (OR, 0.70; 95% CI, 0.55-0.89; *I*^2^ = 69.41%; aOR, 0.73; 95% CI, 0.63-0.86; *I*^2^ = 13.64%).

**Conclusion and Relevance:**

In this systematic review and meta-analysis of vaccination coverage among forcibly displaced, pediatric populations, vaccination coverage remained low. These findings highlight the urgent need for development of healthcare strategies and policies to close the immunization gap in this vulnerable population.

## Key Points

**Question:**

What is the vaccination coverage among forcibly displaced, pediatric populations, and what factors are associated with vaccination?

**Findings:**

In this systematic review and meta-analysis of 39 studies from 24 countries, the full vaccination coverage among forcibly displaced, pediatric populations was 21%. Significant factors associated with vaccination included being forcibly displaced, having 2 or more children in the family, guardian education, father's employment, guardian education, father’s employment, household income, housing stability, and area of residence.

**Meaning:**

Vaccination is a fundamental health intervention, yet forcibly displaced, pediatric populations are highly vulnerable to underimmunization; this study identified multiple modifiable factors associated with vaccination and targets for intervention.

## Teaser

This systematic review and meta-analysis estimates vaccination coverage and identifies factors associated with vaccination in forcibly displaced, pediatric populations from 24 countries.

## Introduction

As of 2024, the number of forcibly displaced individuals surpassed 120 million.^1^ Although they account for 30% of the global population, children disproportionately comprise 40% of the world’s displaced individuals.^1^ Despite global efforts, the number of displaced children has doubled in the last decade.^2^ More than one-half of the world’s unimmunized children live in 31 countries characterized by fragile, conflict-affected, and vulnerable settings that heighten the risk for contracting vaccine-preventable diseases (VPD).^3^ For example, just 16 conflict-affected countries—12% of the global population—accounted for 67% of the world’s polio cases between 2010 and 2015.^4^

The Immunization Agenda 2030 aims to ensure immunization for all, but conflicts around the globe hinder realization of this goal.^5^ Recent outbreaks illustrate the impact of conflict on vaccination. Despite having been eradicated from the region 25 years ago, polio was recently detected in Gaza,^6^ and a measles outbreak in South Sudan claimed the lives of 1200 refugee children younger than 5 years, one-half of whom were unimmunized.^7^

Conflict-related disruptions in health care systems and poor living conditions exacerbate the morbidity and mortality from VPD experienced by forcibly displaced, pediatric populations.^4,8^ Despite the known factors that render this population susceptible to underimmunization, precise vaccination rates remain largely unknown. Additionally, most countries do not include migrants in their national vaccination-coverage data; only 3 countries in the European Union report migrant vaccination coverage in their national data.^9^

Optimizing evidence-based public health strategies to improve vaccination among forcibly displaced, pediatric populations requires a greater understanding of current vaccination coverage and of factors that impact uptake of vaccines. This study aims to assess vaccination coverage among forcibly displaced, pediatric populations and to identify factors associated with vaccination.

## Methods

This systematic review and meta-analysis was conducted in accordance with the Preferred Reporting Items for Systematic Reviews and Meta-Analyses (PRISMA) reporting guidelines.^10^ The study protocol was registered with PROSPERO (CRD42024521956). Because this study is a systematic review and meta-analysis of previously published literature and did not involve human participants or identifiable personal data, approval by an ethics committee was not required.

### Data Sources

A medical librarian performed comprehensive, systematic searches to identify studies addressing vaccination in forcibly displaced, pediatric populations. Searches of the following databases were conducted in November 2023: Ovid MEDLINE (in-process and other nonindexed citations and Ovid MEDLINE from 1946 to present), Ovid EMBASE (1974 to present), PubMed, Scopus, Web of Science, and Cochrane. The search strategy included all appropriate controlled vocabulary and keywords for *vaccination*, *pediatrics*, and *displaced populations*. The search strategy contained no restrictions for language, publication date, or article type. Full details are available in the eMethods in Supplement 1.

### Study Selection

Included articles presented original research assessing vaccination in populations that were forcibly displaced as defined by the United Nations High Commissioner for Refugees.^11^ Specifically, forcibly displaced populations include refugees, asylum seekers, and internally displaced persons who have been forced to flee their home due to conflict, violence, persecution, or human rights violations. Only pediatric individuals, younger than 19 years, were included (Table 1). Primary research studies, including observational and intervention studies, were included. Systematic reviews, nonoriginal research articles, and qualitative studies were excluded. Each abstract was screened for relevance by 2 independent reviewers (N.K. and C.C.), and the full texts were subsequently screened for inclusion by 2 independent reviewers (N.K. and C.C.). See the eAppendix in Supplement 1 for studies excluded at the full text screening stage. Any conflict was resolved by a third independent reviewer (S.V.). Bibliographies for included studies were scanned, and any relevant studies were screened. Screening was conducted using Covidence.^12^

**Table 1.** PICOS Criteria

PICOS criteria	Inclusion criteria	Exclusion criteria
Population	Forcibly displaced, pediatric populations (<19 y) as defined by United Nations High Commissioner for Refugees	Nonforcibly displaced population or adult population
Interventions and comparators	Any intervention or factor associated with vaccination coverage (eg, health care access, socioeconomic status, educational interventions, or policy changes)	Studies that do not report vaccination coverage rates or do not examine factors associated with vaccination
Outcomes	Vaccination coverage (eg, percentage of population vaccinated) and factors associated with vaccination	Outcomes not associated with vaccination
Study design	Primary research studies, including observational studies (eg, cohort, cross-sectional, or case-control) and interventional studies	Systematic reviews, qualitative research, policy documents, nonoriginal research (eg, editorials or commentaries)
Other	No language or publication date restrictions	Studies not available in full text

Abbreviation: PICOS, population, interventions, comparators, outcomes, and study design.

### Data Extraction

Data from each included study were extracted using an extraction template (eTable 1 in Supplement 1). Two independent reviewers (S.V. and N.K.) extracted data for each article, and a third independent reviewer (C.C or I.I.) resolved any discrepancies. Descriptive statistics and measures of central tendency were used to characterize the extracted continuous variables, and categorical variables were summarized using percentages and frequencies. A study’s authors were contacted if additional data were needed.

The primary outcome was pooled vaccination coverage, defined as the proportion of the target population that was vaccinated. The numbers of vaccinated individuals were treated as events and the total target populations as denominators. The following categories of vaccination coverage were analyzed: full immunization as reported by the studies, coverage after vaccination campaigns, measles-containing vaccine (MCV; 1 or 2 doses as reported), pertussis-containing vaccine (PCV; all 3 doses), polio vaccine (both inactivated polio vaccine and oral polio vaccine), hepatitis B vaccine (all 3 doses), and Bacillus Calmette-Guerin (BCG) vaccine (1 dose) (eTable 2 in Supplement 1). To accommodate regional variations in the accepted standards for full immunization, the definition for full immunization was adopted from each individual study (eTable 2 in Supplement 1).

### Statistical Analysis

#### Main Analysis

A random-effects model using the inverse-variance method was employed to calculate pooled proportions. The logit transformation was used to stabilize the variances. Among the studies evaluating vaccination campaigns, an individual study may report coverages for multiple vaccine types within the same study population. To account for within-study correlations due to repeated observations, a multilevel random-effects model with a random intercept at the study level was employed.

For factors associated with vaccination, unadjusted odds ratios (ORs) or adjusted ORs (aORs) were analyzed in separate meta-analyses. If sufficient data were available, ORs were estimated. For each factor that was reported in 2 or more studies, a meta-analysis—utilizing a random-effects model to account for between-study variability and a logarithmic transformation to stabilize variances—was conducted to determine the pooled ORs and their 95% CIs. The individual aORs included in the pooled aOR were obtained from models that adjusted for different sets of covariates across studies (eTable 3 in Supplement 1).

Heterogeneity across studies was assessed using the *I*^2^ statistic, with predefined thresholds for low (0%-25%), moderate (26%-50%), substantial (51%-75%), and considerable (>75%). Given that high *I*^2^ values are common in meta-analyses of proportions, an influence analysis was conducted. For factors with considerable heterogeneity, leave-one-out sensitivity analysis identified and—in subsequent subanalyses—excluded studies contributing the most to heterogeneity. Publication bias was assessed using traditional funnel plots, alternative funnel plots,^13^ and the Begg test. Compared with traditional funnel plots, alternative funnel plots—which plot study size against log odds—more accurately assess publication bias in meta-analysis of proportions.^13^

All *P* values were 2-sided, and statistical significance was assessed at the .05 α level. To account for multiple hypothesis testing, the Benjamini-Hochberg method was applied.^14^ All statistical analyses were performed using R version 4.4.0 with packages meta version 8.0.2 and metafor version 4.8.0 (R Project for Statistical Computing).

#### Quality and Risk of Bias

First, each included study was critically appraised using the Downs and Black Checklist.^15^ Two reviewers (S.V., N.K., I.I., or N.T.) independently appraised the quality of evidence, resolving discrepancies through discussions and assigned scores with a maximum score of 32. Using the numerical scores, each study was classified as excellent (≥26), good (20-25), fair (15-19), and poor (≤14).^16^

Second, the overall quality of evidence for each pooled outcome was assessed using the Grading of Recommendations Assessment, Development, and Evaluation (GRADE) criteria.^17^ As is recommended for systematic reviews of prevalence, the GRADE approach for overall prognosis was employed to evaluate the evidence on vaccination coverage.^18,19^

## Results

**Figure 1.**
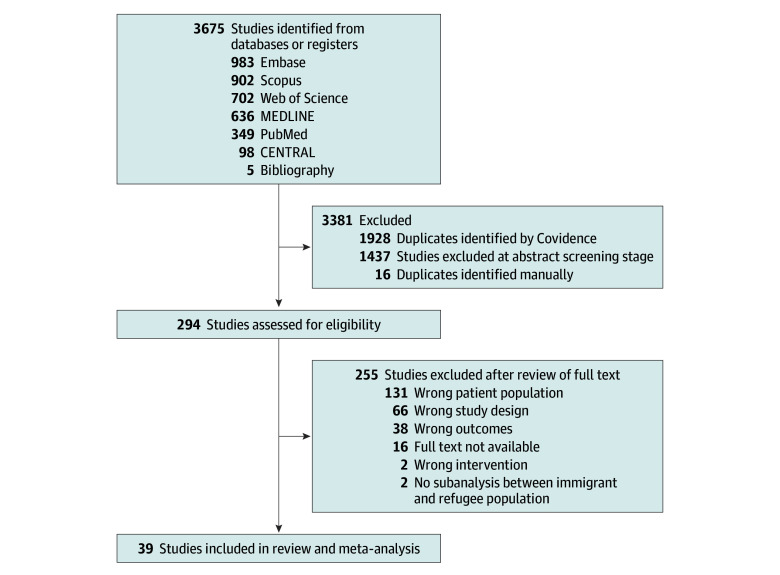
Flowchart of Studies

A total of 1731 unique records were screened (1731 for title or abstract and 294 for full text), of which 39 studies^20,21,22,23,24,25,26,27,28,29,30,31,32,33,34,35,36,37,38,39,40,41,42,43,44,45,46,47,48,49,50,51,52,53,54,55,56,57,58^ were included (Figure 1). The included studies primarily focused on refugees (22 studies^20,21,22,23,24,25,26,27,28,29,30,31,32,33,34,35,36,37,38,39,40,41^) and internally displaced people (8 studies^42,43,44,45,46,47,48,49^). Three studies focused on asylum-seekers,^50,51,52^ and 5 studies^53,54,55,56,57^ included a combination of forcibly displaced populations. Only 1 study^58^ included undocumented individuals. Studies were conducted in 24 countries and had a cross-sectional (23 studies^20,21,26,27,28,30,31,32,33,34,35,36,37,39,40,42,43,46,48,52,53,57,58^), cohort (8 studies^22,23,24,25,51,54,55,56^), or other study design (8 studies^29,38,41,44,45,47,49,50^). Detailed characteristics of the included studies are provided (eTable 4 in Supplement 1).

**Figure 2.**
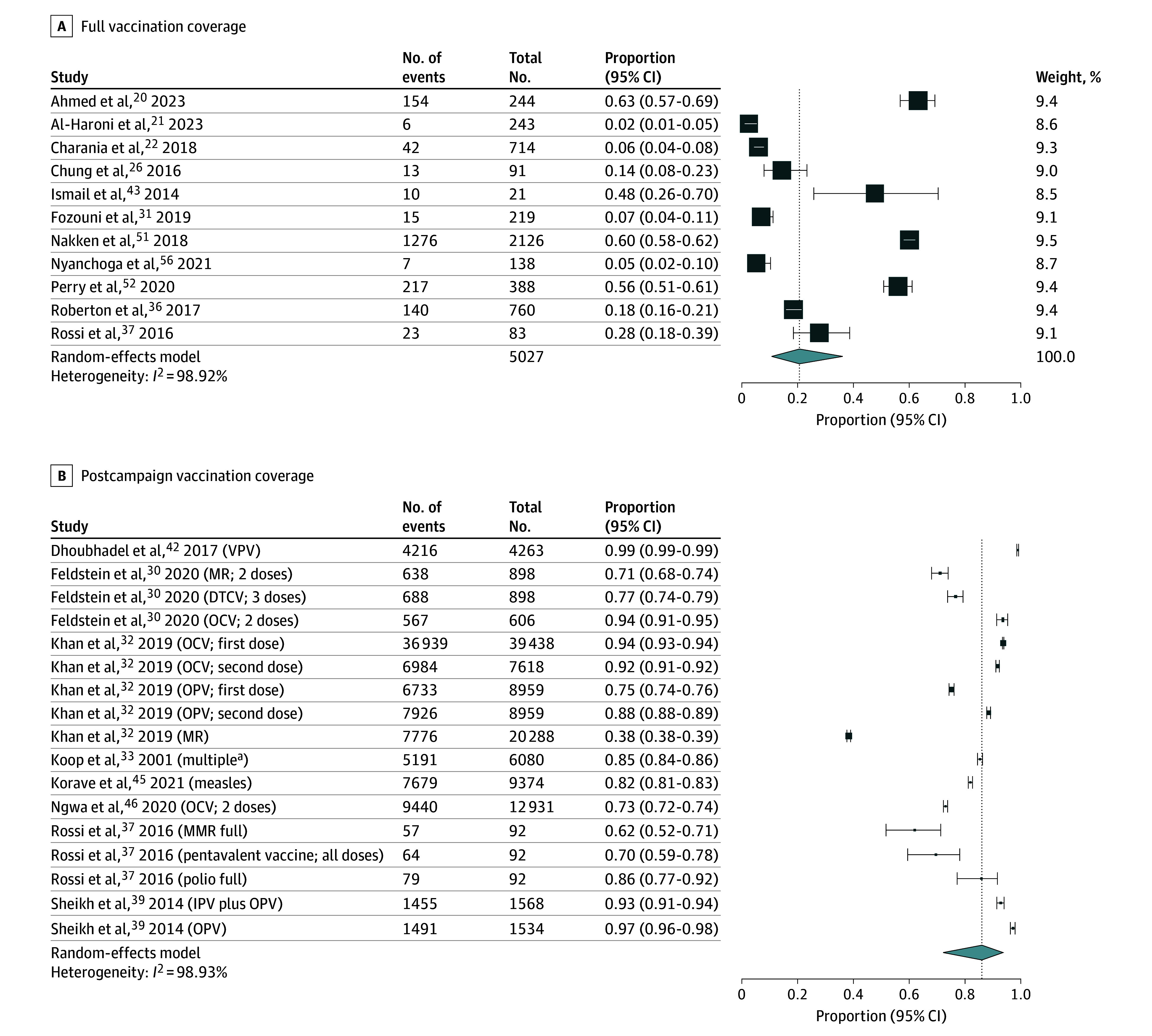
Vaccination Coverage in Forcibly Displaced, Pediatric Populations DTCV indicates diptheria-tetanus containing vaccine; IPV, inactivated polio vaccine; MMR, measles, mumps and rubella; MR, measles and rubella vaccine; OCV, oral cholera vaccine; OPV, oral polio vaccine; VPV vi polysaccharide vaccine for typhoid. ^a^Bacillus Calmette-Guérin; diphtheria, tetanus, and pertussis; OPV; and MMR

Vaccination coverage was reported in 31 studies.^20,21,22,23,24,25,26,27,30,31,32,33,34,35,36,37,39,40,41,42,43,45,46,47,48,49,51,52,53,54,56^ The pooled coverage for full immunization, based on data from 11 studies^20,21,22,26,31,36,37,43,51,52,56^ encompassing 5027 individuals, was 21% (95% CI, 11%-36%; *I*^2^ = 98.92%) (Figure 2A). Coverage after vaccination campaign, analyzed from 8 studies^30,32,33,37,39,42,45,46^ with a total population of 123 690 individuals, was higher at 86% (95% CI, 72%-94%; *I*^2^ = 98.93%) (Figure 2B). Analysis of specific vaccines showed the following rates. MCV, based on data from 10 studies^21,25,26,27,34,35,36,37,52,54^ with a population of 12 869 individuals, was 47% (95% CI, 31%-63%; *I*^2^ = 99.58%). PCV, from 9 studies^21,25,26,27,34,35,36,37,54^ with 18 845 individuals, was 28% (95% CI, 16%-44%; *I*^2^ = 99.72%). Polio vaccine, from 7 studies^26,27,34,35,36,37,53^ with 10 037 individuals, was 29% (95% CI, 13%-53%; *I*^2^ = 99.72%). Hepatitis B vaccine, from 6 studies^21,26,27,34,35,41^ with 10 260 individuals, was 38% (95% CI, 17%-64%; *I*^2^ = 99.72%); and BCG, from 5 studies^21,26,30,35,36^ with 4812 individuals, was 64% (95% CI, 48%-77%; *I*^2^ = 98.72%) (eFigure 1 in Supplement 1).

Vaccination was found to be significantly associated with several factors (Table 2). Factors associated with greater odds of vaccination included having 2 or more children in the family (pooled OR, 1.70; 95% CI, 1.43-2.02; *I*^2^ = 0.00%), guardian having at least some education (pooled OR, 1.60; 95% CI, 1.06-2.43; *I*^2^ = 71.60%), father having employment (pooled OR, 2.75; 95% CI, 1.67-4.53; *I*^2^ = 0.00%), stable housing (pooled OR, 2.80; 95% CI, 2.10-3.73; *I*^2^ = 0.00%), and having access to more health care services (pooled OR, 2.11; 95% CI, 1.44-3.09; *I*^2^ = 0.00%), while being forcibly displaced was associated with lower odds of vaccination (pooled OR, 0.61; 95% CI, 0.44-0.84; *I*^2^ = 91.74%). For adjusted analyses, significant factors associated with higher odds of vaccination included having 2 or more children in the family (pooled aOR, 1.81; 95% CI, 1.28-2.57; *I*^2^ = 0.00%), and stable housing (pooled aOR, 2.62; 95% CI, 1.55-4.40; *I*^2^ = 0.00%), while being forcibly displaced was associated with lower odds of vaccination (pooled aOR, 0.73; 95% CI, 0.63-0.86; *I*^2^ = 13.64%) (Table 3 and eFigure 2 in Supplement 1).

**Table 2.** Factors Associated With Vaccination in Forcibly Displaced, Pediatric Populations

Factors	Studies, No	OR (95% CI)	*P* value	*I*^2^, %
Nationality				
Host nation	NA	1 [Reference]	NA	NA
Displaced population (including Perry et al^52^)^a^	5^34,37,44,52,54^	0.61 (0.44-0.84)	.003	91.74
Displaced population (excluding Perry et al^52^)	4^34,37,44^	0.70 (0.55-0.89)	.004	69.41
Sex of child				
Female	NA	1 [Reference]	NA	NA
Male	4^26,29,37,46^	0.94 (0.81-1.10)	.44	3.50
No. of children in family				
<2	NA	1 [Reference]	NA	NA
≥2	2^25,26^	1.70 (1.43-2.02)	.002	0.00
Age of guardian				
Younger age^b^	NA	1 [Reference]	NA	NA
Older age	2^26,28^	1.42 (0.79-2.55)	.24	0.00
Guardian education				
No education	NA	1 [Reference]	NA	NA
Primary and above	5^20,25,26,28,29^	1.60 (1.06-2.43)	.03	71.60
Father’s employment				
Unemployed	NA	1 [Reference]	NA	NA
Employed	2^20,31^	2.75 (1.67-4.53)	<.001	0.00
Household income				
Low	NA	1 [Reference]	NA	NA
Medium or high (including Charania et al^25^^c^^,^^d^)	5^20,25,26,27,54^	1.10 (0.67-1.81)	.70	77.27
Medium or high (excluding Charania et al^25^)	4^20,26,27,54^	1.33 (1.29-1.37)	<.001	0.00
Housing^e^				
Housing uncertainty	NA	1 [Reference]	NA	NA
Housing stability	3^20,30,37^	2.80 (2.10-3.73)	<.001	0.00
Area of residence^f^				
Normal area	NA	1 [Reference]	NA	NA
Area with better health care services	3^31,37,52^	2.11 (1.44-3.09)	<.001	0.00

Abbreviations: NA, not applicable; OR, odds ratio.

^a^ The study that contributed most to the heterogeneity in the nationality results.

^b^ Current age of guardian was defined as younger than 40 years by Chung et al^26^ and 18 to 34 years by Debela et al.^28^

^c^ For studies with 3 quantiles, the ORs for medium and high were combined, and low served as the reference.

^d^ The study that contributed most to the heterogeneity in the household income results.

^e^ Whereas housing uncertainty refers to living in makeshift settlements or tents and shelters, stability refers to living in registered camps or houses and apartments.

^f^ A normal area was defined as an area with standard immunization services only, while an area with better health care services includes in-house immunization services, primary health care facilities, or part-time health visitors.

**Table 3.** Factors Associated With Vaccination in Forcibly Displaced, Pediatric Populations After Adjusting for Covariates^a^

Factors	Studies, No	aOR (95% CI)	*P* value	I^2^ (%)
Nationality				
Host nation	NA	1 [Reference]	NA	NA
Displaced population	3^34,37,54^	0.73 (0.63-0.86)	<.001	13.64
Sex of child				
Female	NA	1 [Reference]	NA	NA
Male	5^26,32,37,46,51^	1.04 (0.92-1.18)	.52	55.11
No. of children in family				
<2	NA	1 [Reference]	NA	NA
≥2	2^25,26^	1.81 (1.28-2.57)	.001	0.00
Guardian education				
No education	NA	1 [Reference]	NA	NA
Primary and above	3^20,25,26^	1.25 (0.95-1.65)	.11	0.00
Housing^b^				
Housing uncertainty	NA	1 [Reference]	NA	NA
Housing stability	2^20,37^	2.62 (1.55-4.40)	<.001	0.00

Abbreviations: aOR, adjusted odds ratio; NA, not applicable.

^a^ See eTable 3 in Supplement 1 for a list of covariates for each study.

^b^ Whereas housing uncertainty refers to living in makeshift settlements or tents and shelters, stability refers to living in registered camps or houses and apartments.

An influence analysis was conducted for each vaccination coverage outcome (eFigure 3 in Supplement 1). Because fewer than 10 studies reported results for the other vaccine categories, the publication-bias analysis was limited to full vaccination and MCV. Using the Begg test, no publication bias was detected for full vaccination (*z* = −0.70; *P* = .48) or for MCV (*z* = −0.27; *P* = .79) (eFigure 4 in Supplement 1).

For factors with considerable heterogeneity, a leave-one-out sensitivity analysis was conducted, and the single study contributing the most to heterogeneity was removed (eFigure 5 in Supplement 1). After excluding Perry et al,^52^ the heterogeneity of the remaining studies evaluating forcible displacement decreased to 69.41%, and the updated OR was 0.70 (95% CI, 0.55-0.89). For household income, excluding Charania et al^25^ reduced the heterogeneity from 77.27% to 0.00% and yielded a significant OR of 1.33 (95% CI, 1.29-1.37) (Table 3).

Because methods for detecting publication bias require at least 10 studies, the limited number of studies available for each factor did not permit assessment of publication bias. Additionally, all significant findings remained significant after applying the Benjamini-Hochberg method (eTable 5 in Supplement 1).

Based on quantitative data from 4 studies,^30,32,39,46^ the primary barriers to vaccination during campaigns were a lack of awareness about the campaign and being unaware of the timing or location of the campaign. Travel difficulties also frequently hindered vaccination uptake (eTable 6 in Supplement 1). Assessing a campaign’s effectiveness and implementing multidose vaccines was made challenging by the regular influx and efflux of displaced populations. Community engagement facilitated vaccination campaigns because community leaders helped mobilize the community to get vaccinated (eTable 7 in Supplement 1).

The quality of the included studies was rated as good for 11 studies (28.2%),^29,37,41,44,46,48,49,52,53,54,56^ fair for 21 studies (53.8%),^20,21,23,24,25,26,27,28,30,31,32,34,35,36,38,43,47,50,51,55,57^ and poor for 7 studies (18.0%)^22,33,39,40,42,45,58^ (eTable 8 in Supplement 1). According to the GRADE criteria, evidence for vaccination coverage for full immunization and for individual vaccines—such as MCV, PCV, polio, hepatitis B, and BCG—was of medium quality, and the overall quality of evidence for vaccination campaigns was low. Evidence quality was high for housing-related factors; medium for factors such as the sex of the child, father’s employment, number of children in the family, and area of residence; and very low for factors including nationality, guardian’s education, age of the guardian, and household income (eTable 9 in Supplement 1).

## Discussion

In this systematic review and meta-analysis, we found that forcibly displaced, pediatric populations had a full vaccination coverage of 21%. Compared with the host nations’ pediatric populations, the forcibly displaced population had significantly lower odds of vaccination (OR, 0.70; aOR, 0.73). Although coverage after vaccination campaigns was high at 86%, the overall full immunization coverage remained insufficient, indicating that routine immunization services can be strengthened to achieve comprehensive coverage in this vulnerable group. Additionally, economic stability—evaluated through father’s employment, household income, and housing stability—was associated with an increased odds of vaccination among displaced populations. Interventions aimed at improving refugee health—like vaccination—almost exclusively focus on medical factors.^59^ Our findings highlight the necessity of addressing modifiable social factors beyond health factors to more effectively address the immunization challenges faced by displaced populations.

Many countries fall short of the 90% immunization coverage benchmark proposed by the World Health Organization. Afghanistan, for example, reports the lowest full immunization coverage in South Asia at 42.6%.^60^ National disparities are evident, with full immunization coverage across 25 sub-Saharan African countries ranging from 93% in Rwanda to 24% in Guinea.^61^ Disparities are further pronounced among disadvantaged populations; in Uganda, 1 district reported an overall immunization rate of 51%, but lower coverage prevailed in urban slums (48.9%) and rural areas (43.2%).^62^ Our findings reveal that full immunization coverage in forcibly displaced, pediatric populations—at just 21%—is much lower than that reported in other disadvantaged groups. Additionally, we found that forcibly displaced children had approximately 30% lower odds of being vaccinated compared with those from the host nations. Multiple studies identified challenges to obtaining vaccination that are faced by forcibly displaced populations, including barriers arising from restrictive policies, organizational limitations, community standards, intrapersonal relationships, and personal factors.^63,64,65,66,67,68^ In addition to filling a critical gap in knowledge, our findings make evident that profound barriers contribute to low vaccination coverage among forcibly displaced, pediatric populations.

In forcibly displaced populations, vaccination campaigns are commonly implemented to increase coverage and control epidemics. We found postcampaign coverage was 86%. Outbreaks of VPD despite the high postcampaign coverage nevertheless reveal significant immunization gaps.^69^ Notably, a study found that cholera, measles, and poliomyelitis—diseases requiring multidose vaccines—were the most common outbreaks affecting displaced individuals.^70^ Our analysis revealed lower coverage rates for vaccines requiring multiple doses, including PCV, MCV, polio vaccines, and hepatitis B vaccines. This pattern aligns with findings from other studies that report highest coverage for the first vaccine dose, followed by a significant drop with each additional dose.^71,72,73,74^ Because they are frequently encountered while in transit, it is challenging to ensure that displaced individuals complete the entire vaccination series, making high dropout rates common for vaccines requiring multiple doses.^75,76^ Additionally, cold chain requirements, funding shortages, and supply chain limitations pose further barriers for delivering some vaccines.^65^ These barriers may reduce the feasibility of delivering certain vaccines specifically in the context of displacement, thereby decreasing both vaccine-specific coverage and the full immunization rate. Examining both the full immunization and vaccine-specific coverage rates is essential for elucidating the vaccine- and population-specific barriers impeding coverage in this population.

Conflict and humanitarian settings require flexible and context-adapted vaccination strategies.^77^ To overcome the logistical challenges this population faces, clinics designed around the fixed and roving multimodal approach show promise.^33,39^ A study identified the combination of fixed sites and multiple mobile service delivery modes as the most effective strategy for improving vaccination outcomes.^78^ Considering that displaced persons have limited opportunities to obtain vaccines, the coadministration of multiple vaccines during the same visit may also enhance campaign effectiveness and increase coverage.

Engaging community leaders was a frequently reported facilitator of campaigns. Their involvement fosters trust, improves awareness, and helps address community sensitivities, which is critical for building long-term acceptance of vaccines.^79,80^ Although ad hoc vaccination campaigns can effectively reduce transmission and increase short-term coverage, integrating routine vaccination services with scalable, community-informed strategies is essential to achieving sustainable immunization coverage and preventing outbreaks.^81,82,83,84^

Social determinants of health (SDOH) play a role in health care access, outcomes, and overall well-being.^85^ Immigration—recognized as an SDOH—influences multiple aspects of health.^86^ Our analysis demonstrated that key economic factors—a core component of SDOH—were significantly associated with vaccination. The odds of being vaccinated were 2.75 times higher if the father was employed, aligning with a finding in nondisplaced children living in urban slums, where paternal employment was associated with increased odds of vaccination (aOR, 1.53).^87^ Furthermore, higher household income was associated with increased odds of vaccination (OR, 1.33), consistent with a study that reported 27% less likelihood of full vaccination among lower wealth quintiles in low- and middle-income countries.^88^ Additionally, housing instability can adversely affect vaccination and health-intervention rates.^63,89^ Indeed, we found increased odds of vaccination among individuals with stable housing (OR, 2.80; aOR, 2.62). Economic stability has been shown to affect health outcomes in other vulnerable groups. Among forcibly displaced populations, displacement magnifies the negative health impacts of economic instability. Health equity cannot be achieved in this population without addressing the modifiable factors that are vital for economic stability.

Low vaccination coverage is correlated with lower rates of seeking medical care, obtaining antenatal care, delivering in an institution, and utilizing other public health interventions.^90^ Our analysis revealed 2.11 times higher odds of vaccination for displaced populations residing in areas with enhanced health care services—availability of in-house immunization services, primary health care facilities, and part-time health visitors––compared with those in resource-deficient regions.^31,37,52^ At least in areas with enhanced services, health care resources should be leveraged to help address disparities in vaccination. Addressing vaccination gaps can potentially serve as a gateway for improving overall health care engagement in this vulnerable population.

We additionally identified studies that investigated a variety of scalable strategies—including robust monitoring, conducting health promotion and education campaigns, integrating immunization services into nutrition sites, and implementing digital technology—that enhanced vaccination rates, caregiver knowledge, and timeliness of visits for vaccination.^29,31,38,44,47,49^ Notwithstanding the incremental improvements they provide, the impact of these innovations remains limited in settings where fundamental health care services are inadequate. Supplemental strategies must be integrated into a strengthened health care infrastructure to maximize their efficacy. Together, robust primary health care systems and complementary innovations are essential for achieving sustainable improvements in vaccination coverage, for advancing progress toward Immunization Agenda 2030, and for promoting health equity even in the most challenging contexts.

### Limitations

The findings should be interpreted while accounting for the study’s limitations, as well as key gaps in the field. First, by focusing primarily on refugees, internally displaced people and asylum seekers are potentially underrepresented, curtailing the overall generalizability of the findings. Second, owing to the heterogeneity of factors assessed across the included studies, pooled analyses could be performed for only a limited set of variables. Future research should explore broader contextual factors (eg, cold chain challenges, type of displacement, and governance structure of the host country) associated with immunization in this population. Third, the varied methodologies of data collection and reporting—like the implementation of diverse scoring systems, binary responses to assess vaccine knowledge, and nonuniform distributions of age across studies—limited the assessment of factors associated with vaccination coverage despite being reported by more than 2 studies. Numerous factors require further attention in subsequent studies (eTable 10 in Supplement 1). Fourth, partial immunization was inconsistently reported, limiting its interpretation as an outcome. In our pooled analysis, pediatric populations who were partially vaccinated were classified as not fully immunized. Fifth, in our analysis of factors, the pooled ORs may be biased by the effect of known confounders, and our estimates for the pooled aORs are likely impacted by the heterogeneity arising from individual studies adjusting for different sets of covariates. Sixth, we were unable to perform a subanalysis based on population types or other demographic variables due to insufficient data. These limitations highlight the need for more standardized data collection in future studies to address vaccination coverage among forcibly displaced, pediatric populations.

## Conclusions

In this systematic review and meta-analysis of vaccination coverage among forcibly displaced, pediatric populations, we found that coverage was consistently low, revealing a substantial gap in health care provision. Vaccination is a fundamental health intervention, yet challenges associated with forced displacement significantly heighten the vulnerability of this group to underimmunization. We identified multiple modifiable factors that could significantly increase vaccination. Our findings provide compelling evidence for the urgent need to strengthen immunization services among forcibly displaced populations, a demographic that has expanded over the past decade due to escalating conflicts.

## Notes

Supplement 1. **eMethods.** Search Strategy **eAppendix.** Studies Excluded at Full-Text Screening Stage **eTable 1.** Extraction Template **eTable 2.** Vaccination Coverage Data Utilised **eTable 3.** Variables Adjusted by Each Study for Adjusted Odds Ratio **eTable 4.** Detailed Characteristics of Included Studies **eFigure 1.** Forest Plot for Vaccination-Specific Coverage **eFigure 2.** Forest Plot for Factors Affecting Vaccination **eFigure 3.** Influence Analysis for Vaccination Coverage **eFigure 4.** Publication Bias for Full Vaccination and Measles Containing Vaccine **eFigure 5.** Leave-One-Out Sensitivity Analysis for Factors With High Heterogeneity **eTable 5.** Multiple Hypothesis Testing **eTable 6.** Quantitative Data for Reasons for Under-vaccination **eTable 7.** Barrier and Facilitator for Vaccination Campaign **eTable 8.** Down and Black Checklist Quality **eTable 9.** GRADE Table **eTable 10.** Factors That Need Further Exploration

Supplement 2.
Data Sharing Statement
