# Supplementary material for: Vaccination in Forcibly Displaced, Pediatric Populations: A Systematic Review and Meta-Analysis
Source: JAMA Netw Open. 2025 Jun 16;8(6):e2516237. doi: 10.1001/jamanetworkopen.2025.16237 (PMC12171943; doi:10.1001/jamanetworkopen.2025.16237)
Supplement: Supplement 1. — eMethods. Search Strategy eAppendix. Studies Excluded at Full-Text Screening Stage eTable 1. Extraction Template eTable 2. Vaccination Coverage Data Utilised eTable 3. Variables Adjusted by Each Study for Adjusted Odds Ratio eTable 4. Detailed Characteristics of Included Studies eFigure 1. Forest Plot for Vaccination-Specific Coverage eFigure 2. Forest Plot for Factors Affecting Vaccination eFigure 3. Influence Analysis for Vaccination Coverage eFigure 4. Publication Bias for Full Vaccination and Measles Containing Vaccine eFigure 5. Leave-One-Out Sensitivity Analysis for Factors With High Heterogeneity eTable 5. Multiple Hypothesis Testing eTable 6. Quantitative Data for Reasons for Under-vaccination eTable 7. Barrier and Facilitator for Vaccination Campaign eTable 8. Down and Black Checklist Quality eTable 9. GRADE Table eTable 10. Factors That Need Further Exploration [file jamanetwopen-e2516237-s001.pdf]

# Supplemental Online Content

Virk S, Milewski A, Khan N, Cheung C, Wright DN, Kaur G. Vaccination in forcibly displaced, pediatric populations: a systematic review and meta-analysis. *JAMA Netw. Open*. 2025;8(6):e2516237. doi:10.1001/jamanetworkopen.2025.16237

**eMethods.** Search Strategy

**eAppendix.** Studies Excluded at Full-Text Screening Stage

**eTable 1.** Extraction Template

**eTable 2.** Vaccination Coverage Data Utilised

**eTable 3.** Variables Adjusted by Each Study for Adjusted Odds Ratio

**eTable 4.** Detailed Characteristics of Included Studies

**eFigure 1.** Forest Plot for Vaccination-Specific Coverage

**eFigure 2.** Forest Plot for Factors Affecting Vaccination

**eFigure 3.** Influence Analysis for Vaccination Coverage

**eFigure 4.** Publication Bias for Full Vaccination and Measles Containing Vaccine

**eFigure 5.** Leave-One-Out Sensitivity Analysis for Factors With High Heterogeneity

**eTable 5.** Multiple Hypothesis Testing

**eTable 6.** Quantitative Data for Reasons for Under-vaccination

**eTable 7.** Barrier and Facilitator for Vaccination Campaign

**eTable 8.** Down and Black Checklist Quality

**eTable 9.** GRADE Table

**eTable 10.** Factors That Need Further Exploration

This supplemental material has been provided by the authors to give readers additional information about their work.

© 2025 Virk S et al. *JAMA Network Open*.

**eMethods. Search strategy.**

**Pubmed (11/16/23)**

("Vaccination"[Mesh] OR vaccin*[tiab]) AND ("Pediatrics"[Mesh] OR "Child"[Mesh] OR child*[tiab] OR pediatric*[tiab] OR adolescen*[tiab]) AND ("Refugees"[Mesh] OR "Transients and Migrants"[Mesh] OR refugee*[tiab] OR migrant*[tiab] OR "Internally displaced" OR asylum*[tiab]) AND (barrier* OR facilitat* OR improve* OR block* OR increase* OR impede*)

**Medline (11/16/23)**

1. exp Vaccination/
2. exp Vaccines/
3. exp Immunization/
4. (vaccin* or immuni*).ti,ab.
5. 1 or 2 or 3 or 4
6. exp Pediatrics/
7. exp Child/
8. exp Infant/
9. exp Adolescent/
10. (child* or pediatric* or paediatric* or adolescen* or minor* or preschool or juvenil* or infan* or teen* or baby or babies or youth or newborn).ti,ab.
11. 6 or 7 or 8 or 9 or 10
12. exp Refugees/
13. exp "Transients and Migrants"/
14. (refugee* or migrant* or asylum* or (displaced adj2 (person* or people* or population*)) or IDP*).ti,ab.
15. 12 or 13 or 14
16. exp Vaccination Refusal/
17. (barrier* or facilitat* or improv* or block* or increase* or impede* or ((positive or negative) adj2 attitude*) or promot* or motiv* or uptake or campaign* or interven* or hesitan* or refus*).ti,ab.
18. 16 or 17
19. 5 and 11 and 15 and 18

**Embase (11/16/23)**

1. exp vaccination/
2. exp vaccine/
3. exp immunization/
4. (vaccin* or immuni*).ti,ab.
5. 1 or 2 or 3 or 4
6. exp pediatrics/
7. exp child/
8. exp infant/
9. exp adolescent/
10. (child* or pediatric* or paediatric* or adolescen* or minor* or preschool or juvenil* or infan* or teen* or baby or babies or youth or newborn).ti,ab.
11. 6 or 7 or 8 or 9 or 10
12. exp refugee/
13. exp migrant/
14. (refugee* or migrant* or asylum* or (displaced adj2 (person* or people* or population*)) or IDP*).ti,ab.
15. 12 or 13 or 14
16. exp vaccination refusal/
17. (barrier* or facilitat* or improv* or block* or increase* or impede* or ((positive or negative) adj2 attitude*) or promot* or motiv* or uptake or campaign* or interven* or hesitan* or refus*).ti,ab.
18. 16 or 17
19. 5 and 11 and 15 and 18

© 2025 Virk S et al. *JAMA Network Open*.

**Cochrane (11/16/23)**

#1 MeSH descriptor: [Vaccines] explode all trees
#2 MeSH descriptor: [Vaccines] explode all trees
#3 MeSH descriptor: [Immunity] explode all trees
#4 vaccin* or immuni*
#5 #1 or #2 or #3 or #4
#6 MeSH descriptor: [Pediatrics] explode all trees
#7 MeSH descriptor: [Child] explode all trees
#8 MeSH descriptor: [Infant] explode all trees
#9 MeSH descriptor: [Adolescent] explode all trees
#10 child* or pediatric* or paediatric* or adolescen* or minor* or preschool or juvenil* or infan* or teen* or baby or babies or youth or newborn
#11 #6 or #7 or #8 or #9 or #10
#12 MeSH descriptor: [Refugees] explode all trees
#13 MeSH descriptor: [Transients and Migrants] explode all trees
#14 refugee* or migrant* or asylum* or (displaced NEAR/2 (person* or people* or population*)) or IDP*
#15 #12 or #13 or #14
#16 MeSH descriptor: [Vaccination Refusal] explode all trees
#17 barrier* or facilitat* or improv* or block* or increase* or impede* or ((positive or negative) NEAR/2 attitude*) or promot* or motiv* or uptake or campaign* or interven* or hesitan* or refus*
#18 #16 or #17
#19 #5 and #11 and #15 and #18

**Scopus (11/16/23)**

( TITLE-ABS-KEY ( barrier* OR facilitat* OR improv* OR block* OR increase* OR impede* OR ( ( positive OR negative ) W/2 attitude* ) OR promot* OR motiv* OR uptake OR campaign* OR interven* OR hesitan* OR refus* ) ) AND ( TITLE-ABS-KEY ( refugee* OR migrant* OR asylum* OR ( displaced W/2 ( person* OR people* OR population* ) ) OR idp* ) ) AND ( TITLE-ABS-KEY ( child* OR pediatric* OR paediatric* OR adolescen* OR minor* OR preschool OR juvenil* OR infan* OR teen* OR baby OR babies OR youth OR newborn ) ) AND ( TITLE-ABS-KEY ( vaccin* OR immuni* ) )

**Web of Science (11/16/23)**

(((ALL=(barrier* or facilitat* or improv* or block* or increase* or impede* or ((positive or negative) attitude*) or promot* or motiv* or uptake or campaign* or interven* or hesitan* or refus*)) AND ALL=(refugee* or migrant* or asylum* or (displaced (person* or people* or population*)) or IDP*)) AND ALL=(child* or pediatric* or paediatric* or adolescen* or minor* or preschool or juvenil* or infan* or teen* or baby or babies or youth or newborn)) AND ALL=(vaccin* or immuni*)

© 2025 Virk S et al. *JAMA Network Open*.

## eTable 1. Extraction Template

| |
|---|
| Covidence number |
| First author |
| Year of publication |
| Type of evidence |
| Study design |
| Aim of study |
| Study location |
| Study setting |
| Country of origin |
| Host nation |
| Type of forcibly displaced population |
| Status of migration |
| Age group of children |
| Vaccine studied |
| Outcome of interest |
| Study population vaccinated fully |
| Study population partially vaccinated |
| Study population not vaccinated |
| Barrier reported |
| Facilitator reported |
| Variables adjusted for |

© 2025 Virk S et al. *JAMA Network Open*.

## eTable2. Vaccination Coverage Data Utilised

### A. Full Immunization (11)

| Author | Study design | Vaccine schedule |
|---|---|---|
| Ahmed et al (2023)[20] | Cross-sectional study | EPI schedule (OCV (Y/N); BCG birth dose (Y/N); Rotavirus (Y/N); Hepatitis birth dose (Y/N); OPV (Y/N); Pentavalent (Y/N); Pneumococcal pneumonia (Y/N); MCV (Measles containing vaccine) (Y/N); Tetanus toxoid (Y/N)) |
| Al-Haroni et al (2023)[21] | Cross-sectional study | Full: 1 dose of BCG; 3 doses of Pentavalent; 3 doses of HepB; 1 dose of MCV; Partial: missed any of the above vaccines or one or more doses; Unimmunised; not recieved a single dose |
| Charania et al (2018)[22] | Retrospective cohort study | Full: complete and timely vaccine of MMR, PCV, and DTP vaccines; No: not complete and timely, includes partially vaccinated, delayed or declined vaccination |
| Chung et al (2016)[26] | Cross-sectional study | 1:3:3:3:1 vaccine schedule (BCG (at 1 month of age): HBV (birth, 1 mo and 6 mo): POL (2, 3, and 4 mo): DTP (3, 4, and 5 mo): MMR (8 mo)) |
| Ismail et al (2014)[43] | Cross-sectional study | Full standard doses of recommended antigens (BCG, Pentavalent, polio and measles vaccines) within the recommended time of less than 1 year of age" |
| Fozouni et al (2019)[31] | Cross-sectional study | 3 polio; 2 measles and 3 tetanus |
| Nakken et al (2018)[51] | Retrospective cohort study | Danish national guidelines |
| Nyanchoga et al (2021)[56] | Retrospective cohort | Australian catch up immunisation (MMR; Polio; DTP; HPV; Hib) |
| Perry et al (2020)[52] | Cross-sectional study | Those aged 5 to 9 years were considered up to date if they had received three doses of DTaP/IPV/Hib (diphtheria, tetanus, inactivated polio, Haemophilus influenzae type b) vaccine, two doses of MMR (Measles, Mumps, Rubella) vaccine and one dose of Hib/ MenC (Haemophilus influenzae type b, Meningococcal serogroup C) vaccine. Those aged 10 to 16 years were con-sidered up to date if they had received three doses of Td/IPV (tetanus, diphtheria, inactivated polio) containing vaccine, two doses of MMR vaccine and one dose of MenACWY (Meningococcal serogroups A, C, W and Y) vaccine. |
| Roberton et al (2017)[36] | Cross-sectional study | Routine vaccination services (having received from non-campaign sources: measles, polio 1–3, and DPT 1–3 in Jordan and Lebanon, and BCG in Jordan). |
| Rossi et al (2016)[37] | Cross-sectional study (pre intervention) | Pre-campaign: Fully vaccinated (MMR, Penta, Polio) |

### B. Coverage after Vaccination Campaign (8)

| Author | Study design | Vaccines delivered |
|---|---|---|
| Dhoubhadel et al (2017)[42] | Cross-sectional study | Vi polysaccharide vaccine |
| Feldstein et al (2020)[30] | Cross-sectional study | MR, DTCV, OCV |
| Khan et al (2019)[32] | Cross-sectional study | OCV, OPV, MR |
| Koop et al (2001)[33] | Cross-sectional study | BCG, DTP, OPV, MMR |
| Korave et al (2021)[45] | Case study | Measles |
| Ngwa et al (2020)[46] | Cross-sectional study | OCV |
| Rossi et al (2016)[37] | Cross-sectional study (post intervention) | MMR +Penta+ Polio |

© 2025 Virk S et al. *JAMA Network Open*.

| | | |
|---|---|---|
| Sheikh et al (2014)[39] | Cross-sectional study | IPV+OPV( december) 6weeks-59 months; OPV (november) <6 weeks |

### C. Measles Containing Vaccine (10)

| Author | Study Design | Vaccine Studied |
|---|---|---|
| Al-Haroni et al (2023)[21] | Cross-sectional study | MMR 1st dose |
| Charania et al (2023)[25] | Retrospective cohort study | Vaccinated if child's age was ≥1 year and <5 years old and the child received at least one dose, or the child was ≥5 years and received two doses (MOH, 2020). Although children <1 year old are not eligible for MMR, we considered them to be age-appropriately vaccinated as they may have received a dose |
| Chung et al (2016)[26] | Cross-sectional study | One dose of MMR (8 months) first 12 months of life |
| Deal et al (2022)[27] | Cross-sectional study | 1-3 years: 1 dose; 3+: 2 doses |
| Mansour et al (2019)[34] | Cross-sectional study | First dose of MCV (card+recall) |
| Moller et al (2016)[54] | Register-based cohort design | MMR1 (15 mo) |
| Perry et al (2020)[52] | Cross-sectional study | Measles two vaccines |
| Öztaş et al (2020)[35] | Cross-sectional study | MMR 1 dose |
| Roberton et al (2017)[36] | Cross-sectional study | MMR |
| Rossi et al (2016)[37] | Cross-sectional study (pre intervention) | MMR1+MMR2 |

### D. Pertussis Containing Vaccine (9)

| Author | Study Design | Vaccine Studied |
|---|---|---|
| Al-Haroni et al (2023)[21] | Cross-sectional study | 3rd dose (DTaP-IPV-/HiB) |
| Charania et al (2023)[25] | Retrospective cohort study | Vaccinated if child received all pertussis-containing vaccines consistent with age and recommended national immunisation schedule or catch-up schedule (MOH, 2020). |
| Chung et al (2016)[26] | Cross-sectional study | Three doses of DTP (3, 4, and 5 months), |
| Deal et al (2022)[27] | Cross-sectional study | Pertussis UK immunisation schedule (2 months – 3 years: 3 doses<br>3 years old: 4 doses) |
| Mansour et al (2019)[34] | Cross-sectional study | DTP 3rd dose |
| Moller et al (2016)[54] | Register-based cohort design | DTaP-IPV |
| Öztaş et al (2020)[35] | Cross-sectional study | 3 doses (DaPT-IPV-Hib) |
| Roberton et al (2017)[36] | Cross-sectional study | DTP 3 doses |
| Rossi et al (2016)[37] | Cross-sectional study (pre intervention) | Pentafull |

© 2025 Virk S et al. *JAMA Network Open*.

## E. Polio Vaccine (7)

| Author | Study Design | Vaccine Studied |
|---|---|---|
| Chung et al (2016)[26] | Cross-sectional study | 3 doses (2, 3, 4 mo) |
| Deal et al (2022)[27] | Cross-sectional study | 2mo - 3 year: 3 doses; 3 years+: 4 doses |
| Rossi et al (2016)[37] | Cross-sectional study (pre-intervention) | Poliofull |
| Gebremedhin et al (2023)[53] | Cross-sectional study | IPV and 3 dose of OPV |
| Mansour et al (2019)[34] | Cross-sectional study | 3rd dose |
| Öztaş et al (2020)[35] | Cross-sectional study | 2 doses OPV |
| Roberton et al (2017)[36] | Cross-sectional study | Polio 3 doses |

## F. Hepatitis B Vaccine (6)

| Author | Study Design | Vaccine Studied |
|---|---|---|
| Al-Haroni et al (2023)[21] | Cross-sectional study | 3rd dose of HepB |
| Chung et al (2016)[26] | Cross-sectional study | 3 doses (birth, 1 mo, and 6 mo) |
| Deal et al (2022)[27] | Cross-sectional study | 2 mo + : 3 doses |
| Mansour et al (2019)[34] | Cross-sectional study | 3rd dose of HepB |
| Öztaş et al (2020)[35] | Cross-sectional study | 3 doses of HepB |
| Yun et al (2016)[41] | Retrospective prevalence study | Serological evidence of vaccination HBV |

## G. BCG Vaccine(5)

| Author | Study Design | Vaccine Studied |
|---|---|---|
| Al-Haroni et al (2023)[21] | Cross-sectional study | One dose |
| Chung et al (2016)[26] | Cross-sectional study | One dose at 1 mo of age |
| Feldstein et al (2020)[30] | Cross-sectional study | Presence of BCG scar |
| Öztaş et al (2020)[35] | Cross-sectional study | One dose |
| Roberton et al (2017)[36] | Cross-sectional study | One dose |

© 2025 Virk S et al. *JAMA Network Open*.

**eTable 3. Variables Adjusted by Each Study for Adjusted Odds Ratio**

| Factors | Study | Variables adjusted for |
|---|---|---|
| Nationality | Mansour et al,[34] 2019 | Sex, age, mother's education, place of last vaccination |
| | Moller et al,[54] 2016 | Equalised by family income |
| | Rossi et al,[37] 2016 | Sex, shelter, age, heard of campaign, living in Middle Dreib, living village |
| Sex of child | Chung et al,[26] 2016 | Variables with univariate p value less than 0.20 (i.e., Child sex, Father's education level, Sibling, Previous miscarriage) were included in each model and retained if their p values were less than 0.05. |
| | Khan et al,[32] 2019 | Multiple logistic regression model was used with appropriate adjustment of covariates. |
| | Nakken et al,[51] 2018 | Country of origin, age group, gender and vaccination status. |
| | Ngwa et al,[46] 2020 | For age group. |
| | Rossi et al,[37] 2016 | Sex, shelter, age, heard of campaign, living in MD, living village |
| Number of children in family | Charania et al,[25] 2023 | Visa category, Age at Arrival, Arrival year, Region, Ethnicity, Dependent children, Household income, Parent education, Parents ability to comprehend English, Family structure |
| | Chung et al,[26] 2016 | Variables with univariate p value less than 0.20 (i.e., Child sex, Father's education level, Sibling, Previous miscarriage) were included in each model and retained if their p values were less than 0.05. |
| Guardian education | Ahmed et al,[20] 2023 | Enter method for adjustment |
| | Charania et al,[25] 2023 | Month and year of birth, month and year of death (if applicable), sex, ethnicity, nationality, visa cetagory, time spent in NZ, number of depenendent children, household income, parent's education, family type, and parent's ability to converse in English. |
| Housing | Ahmed et al,[20] 2023 | Enter method for adjustment |
| | Rossi et al,[37] 2016 | Sex, shelter, age, heard of campaign, living in MD, living village |

© 2025 Virk S et al. *JAMA Network Open*.

## eTable 4. Detailed Characteristics of Included Studies

| Study Author (Year) | Study design | Host Country | Country of Origin | Type of displaced population | Age of children studied | Total: Sample size | Total: Of which pediatric displaced | Outcome: Vaccine studied | Outcome: Other outcome |
|---|---|---|---|---|---|---|---|---|---|
| Ahmed et al (2023)[20] | Cross-sectional study | Bangladesh | Myanmar | Refugee | ≤7 years old | 244 | 244 | Full immunisation: all vaccines in EPI schedule. | Practice of vaccination as confirmed by cross-checking vaccination card |
| Al-Haroni et al (2023)[21] | Cross-sectional study | Malaysia | Myanmar | Refugee | 3-14 years old | 243 | 243 | Full immunisation: 1 dose of BCG vaccine, 3 doses of DTaP/IPV/Hib, 3 doses of HepB and 1 dose of measles. (WHO) | Underimmunisation |
| Charania et al (2018)[22] | Retrospective cohort study | New Zealand | Multiple | Refugee | <18 years old | 75,375 (Cohort A (child's visa)) and 50,136 (Cohort B (mother's visa)) | 837 (Cohort A (child's visa)) and 894 (Cohort B (mother's visa)) | MMR vaccine (2 doses), PCV (3 primary doses and a booster), Pertussis-containing vaccine (3 primary doses and a booster), Rotavirus vaccine (3 doses) | Complete and timely vaccination |
| Charania et al (2023)[23] | Retrospective cohort study | New Zealand | Multiple | Refugee | <18 years old | 847,197 | 8130 (MMR); 8571 (P) | MMR vaccine (2 doses); Pertussis containing vaccine (3 primary doses and 2 boosters); HPV (at least 2 primary doses) | Influence of parental migration on vaccination. |
| Charania et al (2023)[24] | Retrospective cohort study | New Zealand | NA | Refugee | 5-11 years old | 451,323 | 7443 | COVID-19 | |
| Charania et al (2023)[25] | Retrospective cohort study | New Zealand | Multiple | Refugee | <18 years old | 2796 | 2796 | MMR vaccination Pertussis containing vaccination (Vaccinated if | Enrollment in National Immunisation Register |

© 2025 Virk S et al. *JAMA Network Open*.

| | | | | | | | | | |
|---|---|---|---|---|---|---|---|---|---|
| | | | | | | | | child's age was ≥1 year and <5 years old and the child received at least one dose, or the child was ≥5 years and received two doses (MOH, 2020). Although children <1 year old are not eligible for MMR, we considered them to be age-appropriately vaccinated as they may have received a dose.) | |
| Chauhan et al (2019)[50] | Longitudinal interventional study | United Kingdom | Not mentioned | Unaccompanied Asylum Seeker | <18 years old | 14 | 14 | NA | Immunisation status, dental check, eye checkup, Tb screening |
| Chung et al (2016)[26] | Cross-sectional study | China | North korean mother | Refugee | 12 months or older | 91 | 91 | 1:3:3:3:1 series primary immunization (BCG:DTP:HBV:MMR:POL) and VAR and JE | Immunisation rates |
| Deal et al (2022)[27] | Cross-sectional study | United Kingdom | Multiple | Refugee | ≤19 years old | 12526 | 3633 | Polio, MMR, MenACWY | Immunised in accordance with UK immunisation schedule; UK Refugee Technical Instruction (at least one dose) |
| Debela et al (2022)[28] | Cross-sectional study | New Zealand | Multiple | Refugee | 6 week- 16 years old | 178 | 178 | | Vaccine hesitancy |
| Dhoubhadel et al (2017)[42] | Cross-sectional study | Nepal | Nepal | IDP | 2-15 years old | 4263 | 4263 | Typhoid | Vaccination campaign post disaster |
| El-Halabi et al (2023)[29] | Non-randomized controlled trial | Jordan | Syria | Refugee | 0-5 years of age | 936 | 936 | Vaccination Schedule of the Jordan Ministry of Health | Increasing immunization Coverage |

© 2025 Virk S et al. *JAMA Network Open*.

| | | | | | | | | | |
|---|---|---|---|---|---|---|---|---|---|
| Feldstein et al (2020)[30] | Cross-sectional study | Bangladesh | Myanmar | Refugee | ≤14 years of age | 930 | 930 | MR+DTCV+OCV+BCG | Percent of children seroprotected |
| Fozouni et al (2019)[31] | Cross-sectional study | Germany | Multiple | Refugee | 1-5 years of age | 219 | 219 | Fully immunised: full standard doses of BCG, tetanus, polio, and measles | Efficacy of in-camp immunisation services |
| Gebremedhin et al (2023)[53] | Cross-sectional study | Ethiopia | Multiple | IDP + Refugee | 12-35 mo | 3,646 | 575 | OPV (0-3 doses), IPV | Vaccination coverage |
| Ismail et al (2014)[43] | Cross-sectional study | Sudan | Sudan | IDP | 12-23 mo | 213 | 21 | Full standard doses of recommended antigens (BCG, Pentavalent, polio and measles vaccines) within the recommended time of <1 year of age | Immunisation coverage |
| Kaewkungwal et al (2010)[44] | Quasi-experimental study | Thailand | Thai and non-Thai | IDP | ≥18 years old | 544 | 182 | Standard vaccines as determined by Thai government (BCG, DH, DTP, HBV, JE, Measles, OPV) | ANC/EPI coverage |
| Khan et al (2019)[32] | Cross-sectional study | Bangladesh | Myanmar | Refugee | 0-15 | 39,438 | 39,438 | OCV, OPV, MR | OPV vaccination status of 0–5 years children, MR vaccination status of 6 months to less than 15 years old children, as well as one dose OCV coverage status of people aged > 1 year and 2 dose of OCV coverage among children aged 1-<5 years |
| Klok-Nentjes | Cross-sectional study | Netherlands | Multiple | Undocumented | <18 years old | 267 | 39 | | Any childhood vaccination |

© 2025 Virk S et al. *JAMA Network Open*.

| | | | | | | | | | |
|---|---|---|---|---|---|---|---|---|---|
| et al (2018)[58] | | | | | | | | | |
| Koop et al (2001)[33] | Cross-sectional study | Macedonia | Albania | Refugee | <4 years | 102900 | 102900 | BCG, DTP, OPV, MMR | vaccination coverage |
| Korave et al (2021)[45] | Case study | Nigeria | Nigeria | IDP | 9-59 mo | 9374 | 9374 | Measles | vaccination coverage |
| Mansour et al (2019)[34] | Cross-sectional study | Lebanon | Syria | Refugee | 12-59 mo | 9315 | 2179 | HepB(0-3rd dose); Polio(1-3rd dose); DTP(1-3rd dose); Hib(1-3rd dose); MCV (1-2nd dose); RCV (1st dose) | vaccination coverage (card; card+recall); dropout |
| Moller et al (2016)[54] | Register-based cohort design | Denmark | Multiple | Refugee +asylum seeker | <18 years old | 116,907 | 16701 | MMR 2 doses; DTaP-IPV 1 dose | intake of vaccine; child health examination |
| Moller et al(2018)[55] | National cohort | Denmark | Multiple | Refugee +asylum seeker | <18 years old | 22848 | 3264 | HPV | Immunization uptake (ordinary immunisation and catch-up programme) |
| Nakken et al (2018)[51] | Retrospective cohort study | Denmark | Multiple | Asylum-seeker | 3 mo-17 years | 2126 | 2126 | Danish vaccination programme | Vaccination needs |
| Ngwa et al (2020)[46] | Cross-sectional study | Nigeria | Nigeria | IDP | <18 years old | 12931 | 12931 | Cholera | vaccination campaign; crude coverage (complete coverage and atleast one dose; card+recall) |
| Nyanchoga et al (2021)[56] | Retrospective cohort | Australia | Multiple | Refugee +asylum seeker | 0-19 years old | 397 | 138 | MMR, Polio, DTP, HPV, Pneumococcal; Hib | underimmunisation |
| Oladeji et al (2019)[47] | Community based intervention study | South Sudan | South Sudan | IDP | <5 years old | 20,315 (sector 2: total population); 33,379 (sector 5) | 20,315 (sector 2: total population); 33,379 (sector 5) | BCG, OPV, Penta, IPV, Measles | Dropout in vaccination |

© 2025 Virk S et al. *JAMA Network Open*.

| | | | | | | | | | |
|---|---|---|---|---|---|---|---|---|---|
| Öztaş et al (2020)[35] | Cross-sectional study | Turkey | Syria | Refugee | 0-4 years of age | 2,827 | 2,827 | HepB (3); BCG (1); Five component combined (3); CPV (3); OPV (2); MMR (1); Varicella (1); HepA (2) | Republic of Turkey; Ministry of Health's Childhood Vaccine Schedule (not vaccinated; completed vaccine doses; incomplete vaccine doses; number of doses not yet due) |
| Perry et al (2020)[52] | Cross-sectional study | Wales | Not mentioned | Asylum-seeker | 5-16 years old | 56,473 | 388 | Measles; Tetanus; MenC | 5-9 up to date: if recived 3 doses of DTaP/IPV/Hib, 2 doses of MMR, 1 dose of Hib/MenC. 10-16 up to date: 3 doses of Td/IPV, 2 doses of MMR, 1 dose of MenACWY |
| Roberton et al (2017)[36] | Cross-sectional study | Lebanon+ Jordan | Syria | Refugee | 12-23 mo | 760 | 760 | Measles; BCG; Polio; DPT; Full immunisation EPI | location of vaccination; difficulties with vaccination |
| Rossi et al (2016)[37] | Cross-sectional study (pre-post intervention) | Lebanon | Syria | Refugee | 12-59 mo | 210 | 83 (pre-campaign); 92 (post-campaign) | Pentavalent (DTP+ Hib+ HepB), MMR, Polio | fully vaccinated: recived all doses for his/her age according to the Ministry of Public Health vaccination calendar |
| Ruiz-Rodríguez et al (2008)[48] | Cross-sectional study | Colombia | Colombia | IDP | <5 years of age | 369 | 185 | HepB; Hib; DPT; Triple Viral; Polio; BCG | vaccination coverage and relationship with insurance |
| Seal et al (2023)[49] | Randomized controlled trial | Somalia | Somalia | IDP | 0-59 mo | 1269 | 1269 | Measles vaccination, Penta series completion, Timely vaccination | mother/caregiver’s knowledge of child health; and preference for getting their children vaccinate |
| Sheikh et al (2009)[38] | Descriptive epidemo | Australia | Sub-Saharan Africa | Refugee | mean age 12 | 112 | 97 | | clinic attendance (for vaccination) |

© 2025 Virk S et al. *JAMA Network Open*.

| | | | | | | | | | |
|---|---|---|---|---|---|---|---|---|---|
| | logical study and survey | | | | | | | | |
| Sheikh et al (2014)[39] | Cross-sectional study | Kenya | Somalia | Refugee | <=59 mo | 120,196 | 98,365 | IPV and OPV | vaccination campaign coverage |
| Shiferie et al (2023)[57] | Cross-sectional study | Ethiopia | Multiple | IDP + Refugee | 12-35 mo | 3,646 | 3646 | BCG; Penta (1 and 3); MCV (1 and 2) | Vaccination dropout |
| Troiano et al (2022)[40] | Cross-sectional study | Italy | Ukraine | Refugee | <18 years old | 79 | 79 | Hexavalent (DTaP-HepB-IPV-Hib); Men ACWY; Men C; Men B; MMR; Chickenpox; PCV 13; Rotavirus; Hep B; dTp-Polio; HPV; Polio | acceptance or refusal of each vaccine offered, as reported by caregiver. |
| Yun et al (2016)[41] | Retrospective prevelance study | United States of America | Multiple | Refugee | <19 years old | 937 (pre-EPI), 1354 (post-EPI) | 937 (pre-EPI), 1354 (post-EPI) | HepB vaccine | serological evidence of HBV immunisation (negative HBs Ag, positive anti-HBs, negative anti-HBc) |

© 2025 Virk S et al. *JAMA Network Open*.

**eAppendix. Studies Excluded at Full Text Screening Stage**

247 studies excluded after full-text screening.

- 130 had wrong patient population
- 57 had wrong study design
- 39 had wrong outcomes
- 16 studies had no full text available
- 2 studies did no sub analysis between immigrant and refugee population
- 2 studies had wrong intervention
- 1 study had adult population

© 2025 Virk S et al. *JAMA Network Open*.

**eFigure 1. Forest Plot for Vaccination-Specific Coverage**

**A. Measles-Containing Vaccine Coverage**

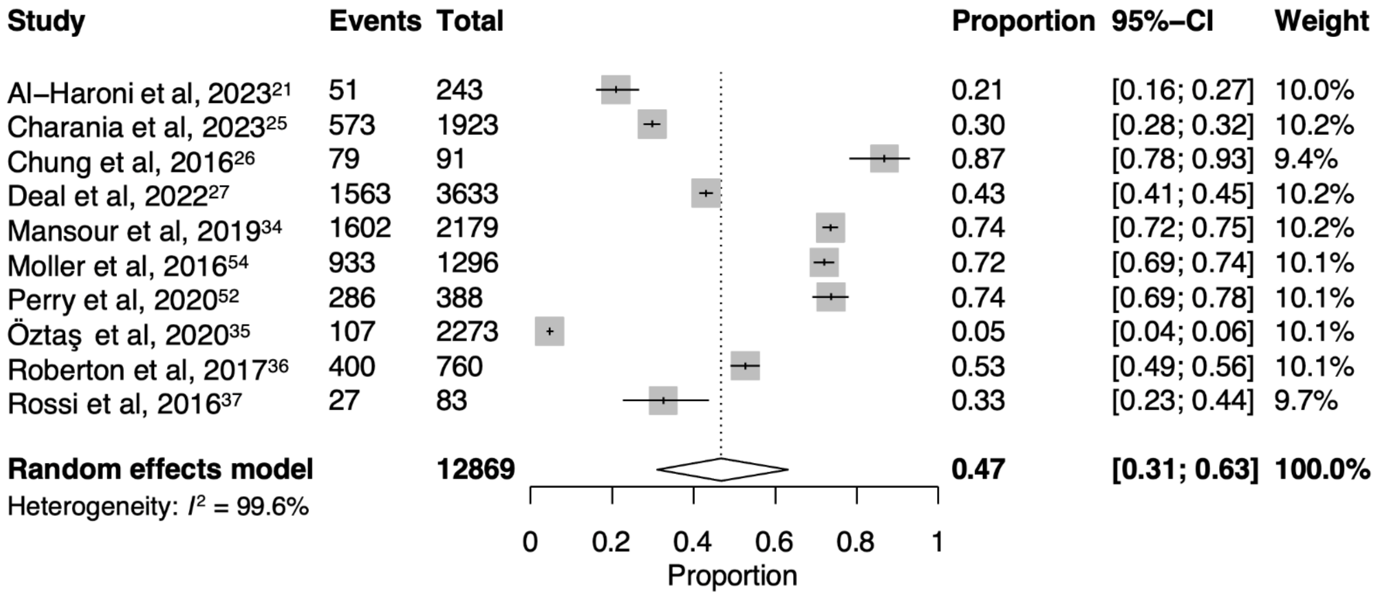


**B. Pertussis-Containing Vaccine Coverage**

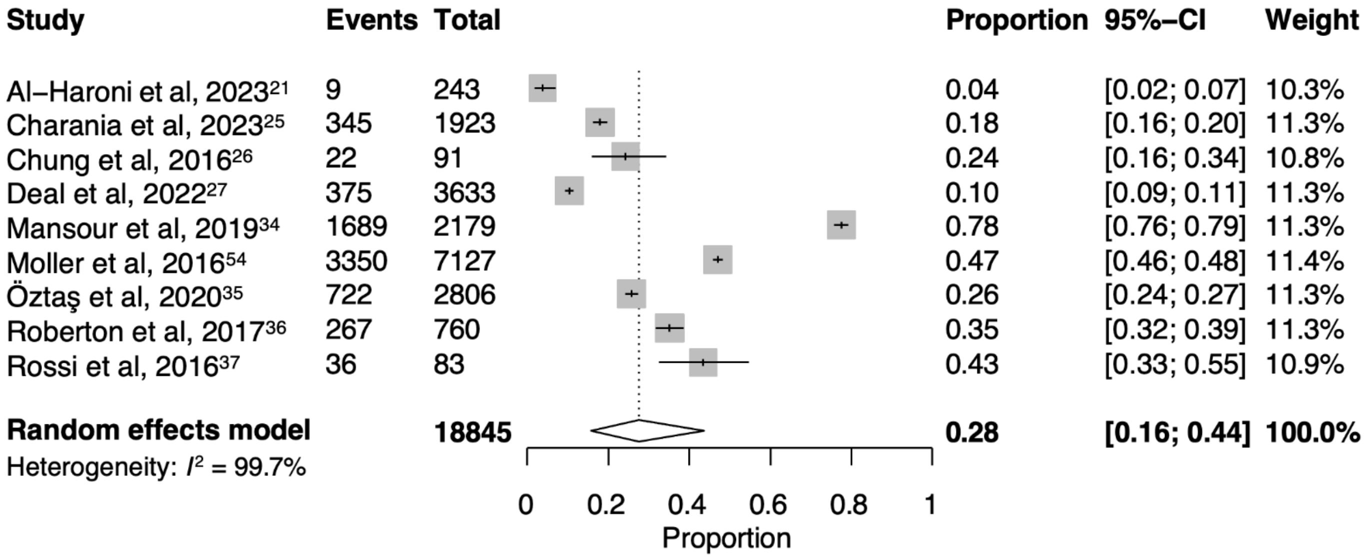


© 2025 Virk S et al. *JAMA Network Open*.

## C. Polio Vaccine Coverage

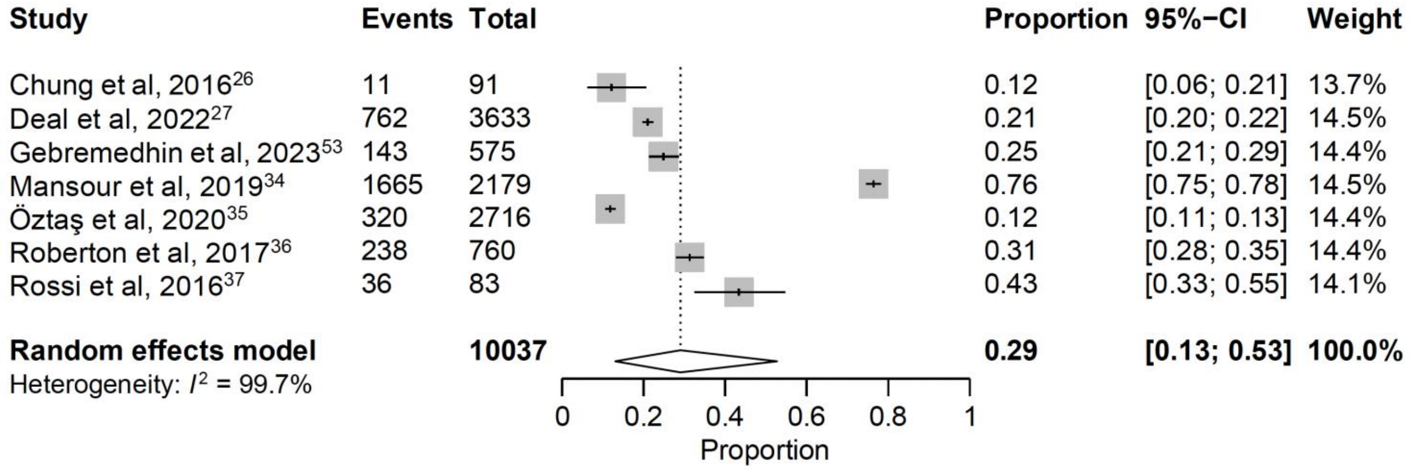


## D. Hepatitis B Vaccine Coverage

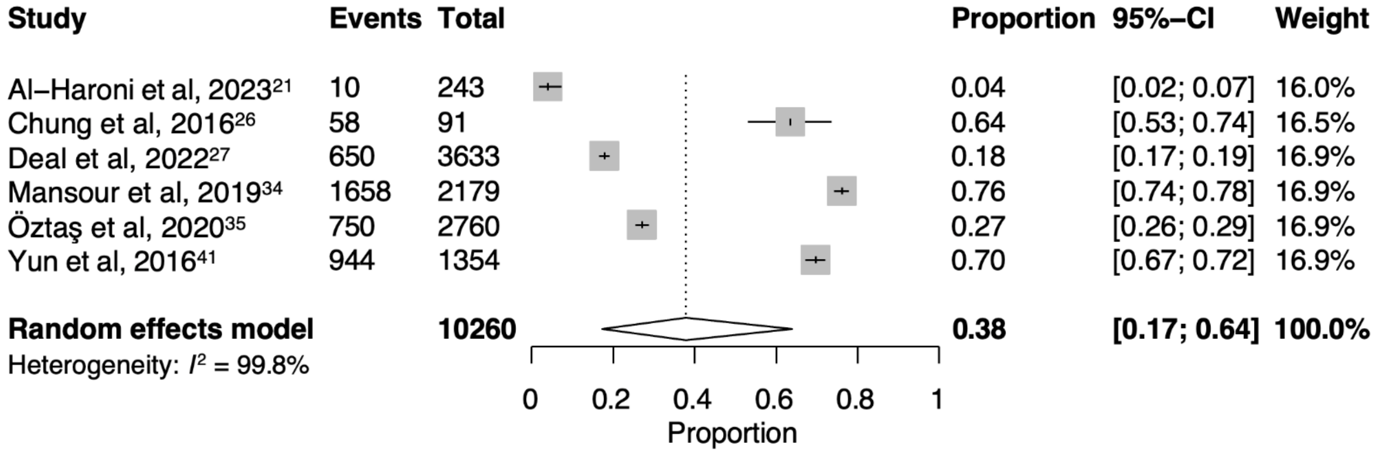


## E. BCG Vaccine Coverage

© 2025 Virk S et al. *JAMA Network Open*.

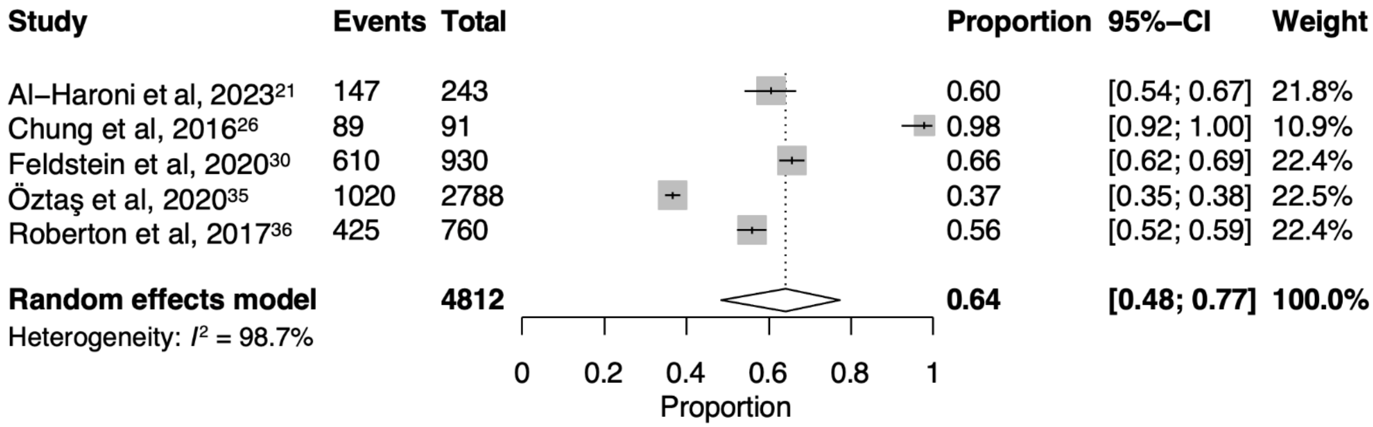


© 2025 Virk S et al. *JAMA Network Open*.

## eFigure 2. Forest plot for factors affecting vaccination

A. **Unadjusted**

i. **Nationality**

a. **Including Perry**

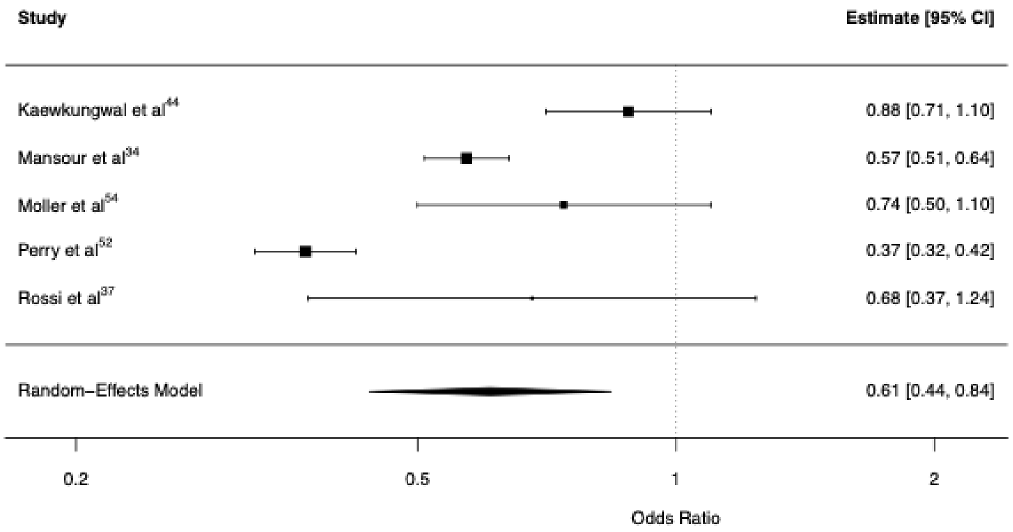


**b. Excluding Perry**

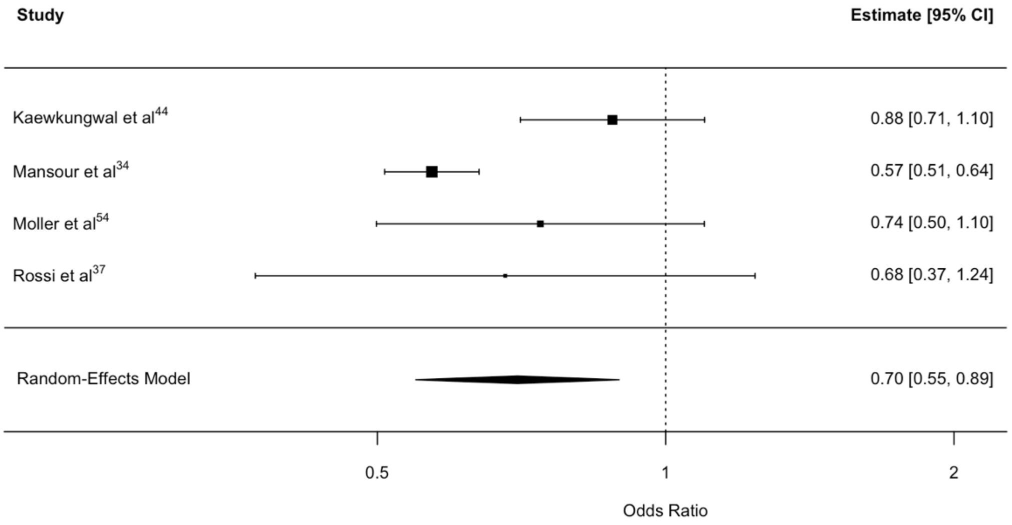


© 2025 Virk S et al. *JAMA Network Open*.

ii. Sex of child

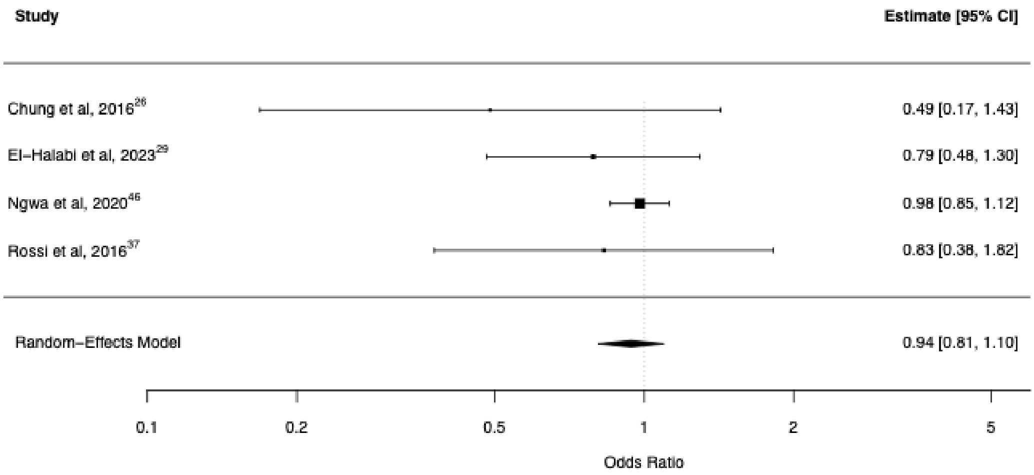


iii. Number of children in family

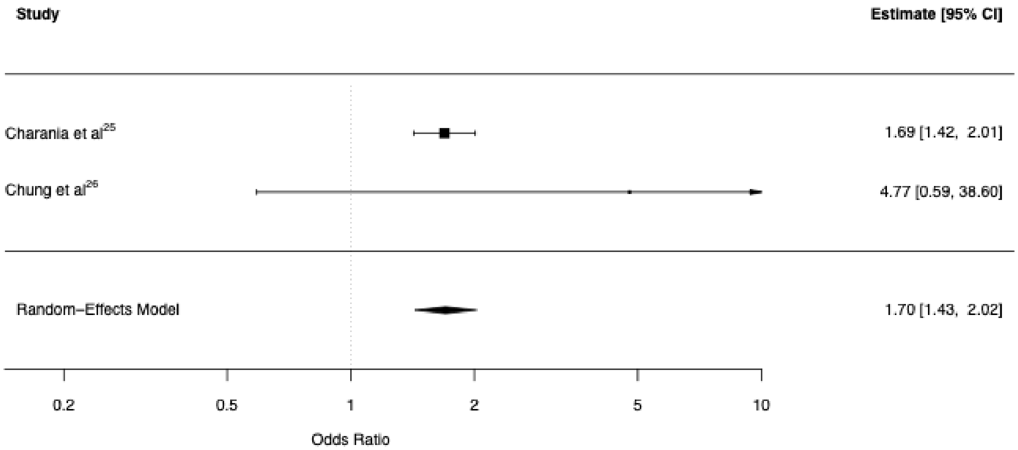


© 2025 Virk S et al. *JAMA Network Open*.

### iv. Age of guardian

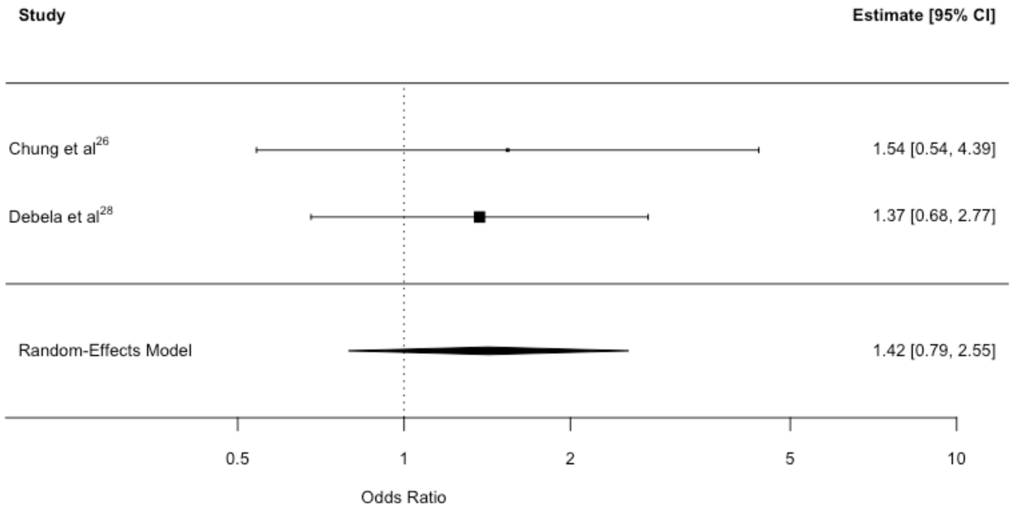


© 2025 Virk S et al. *JAMA Network Open*.

v. **Guardian education**

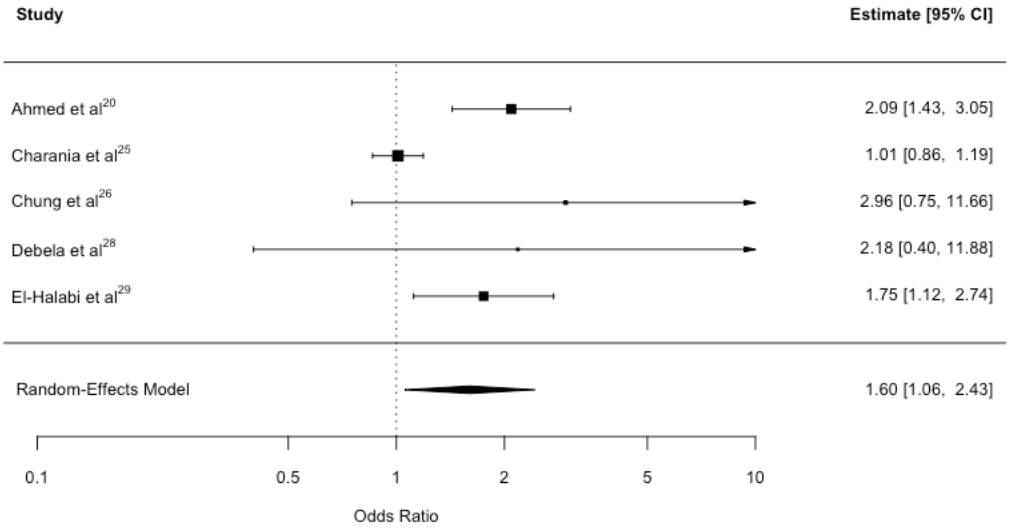


vi. **Father's employment**

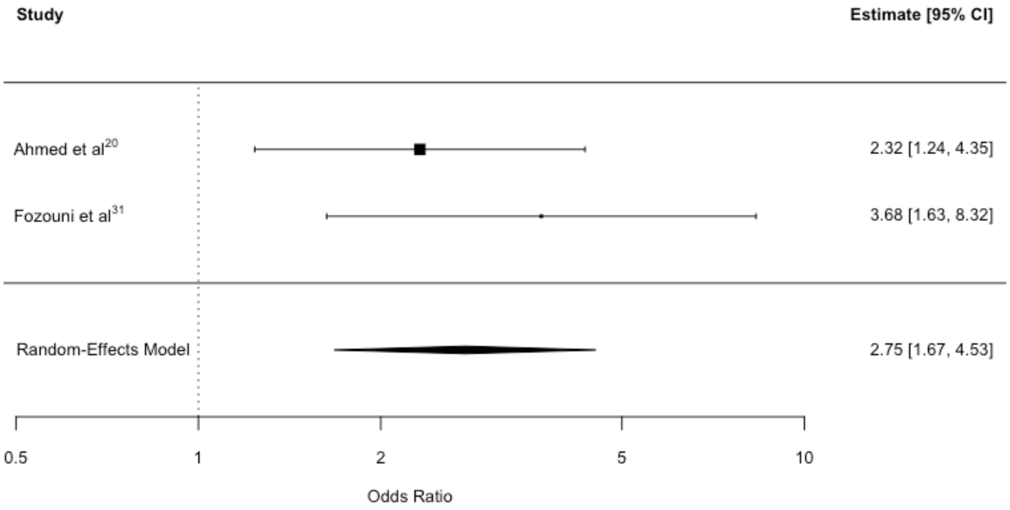


© 2025 Virk S et al. *JAMA Network Open*.

vii. **Household income**

a. **Not excluding Charania**

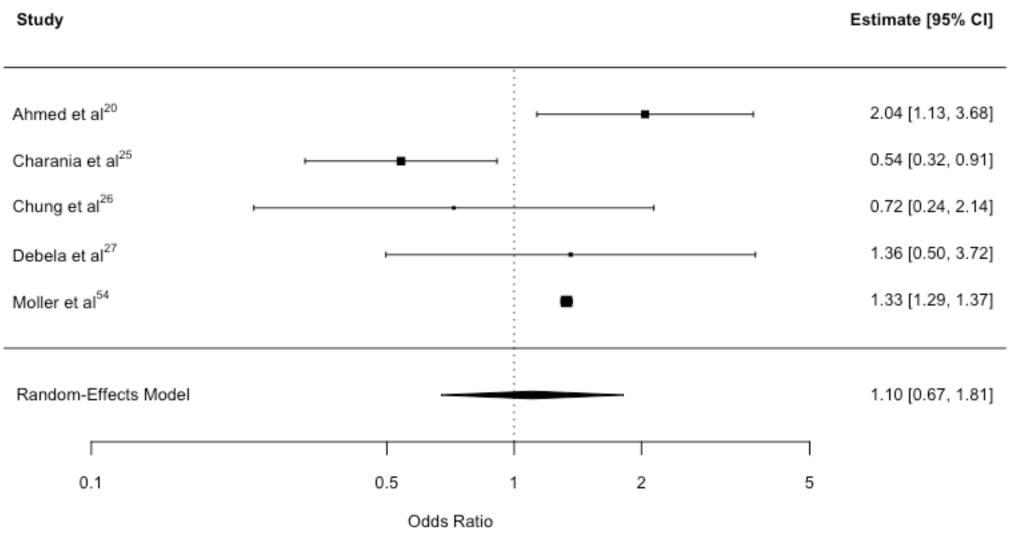


b. **Excluding Charania**

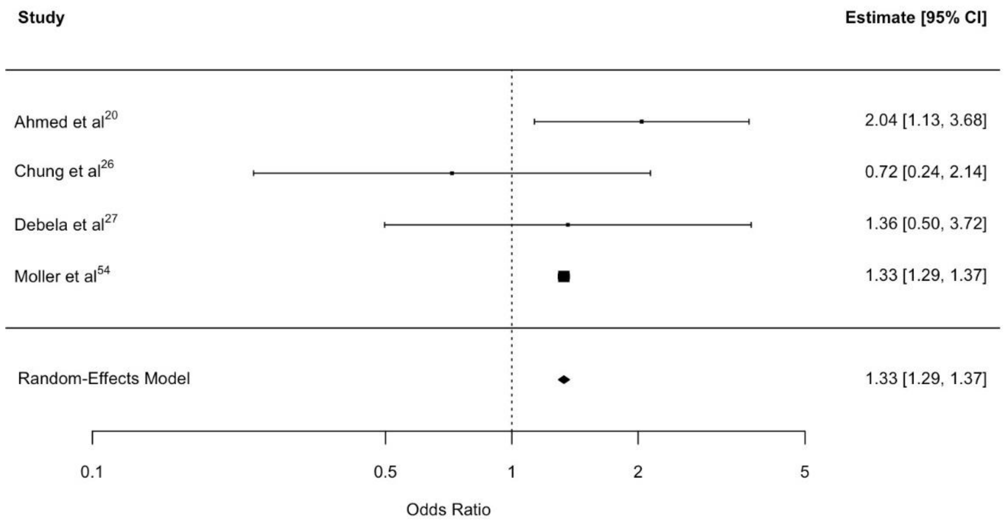


© 2025 Virk S et al. *JAMA Network Open*.

**viii. Housing**

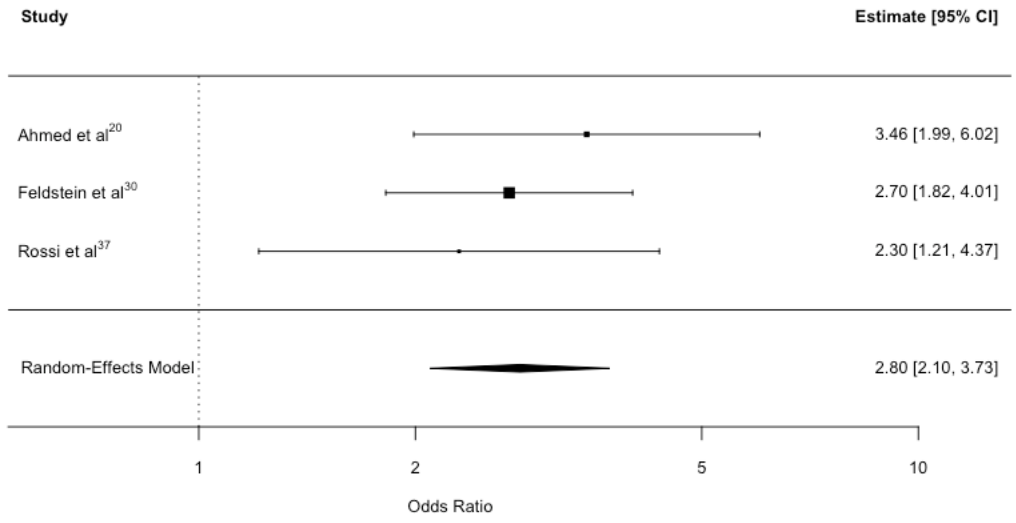


**ix. Area of residence**

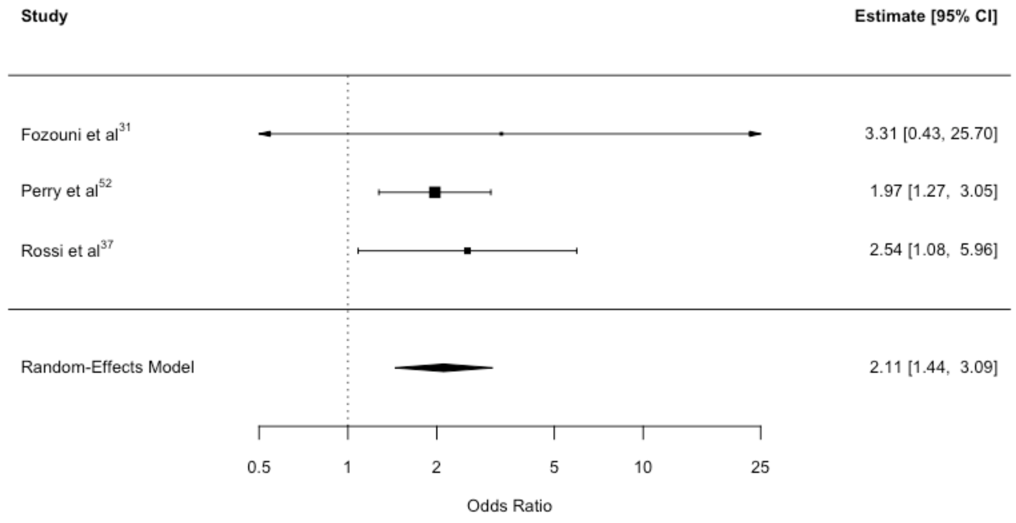


© 2025 Virk S et al. *JAMA Network Open*.

**B. Adjusted**

**i. Nationality**

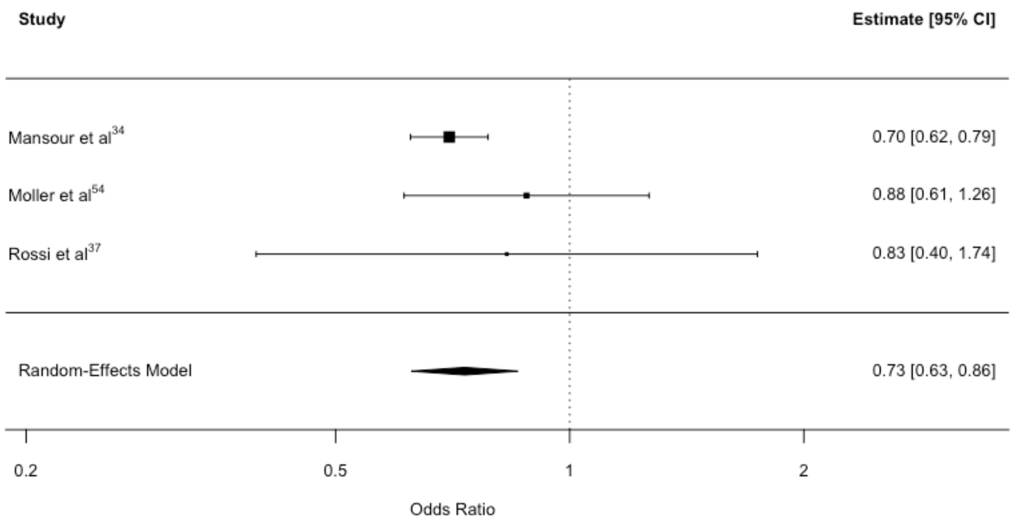


**ii. Sex of child**

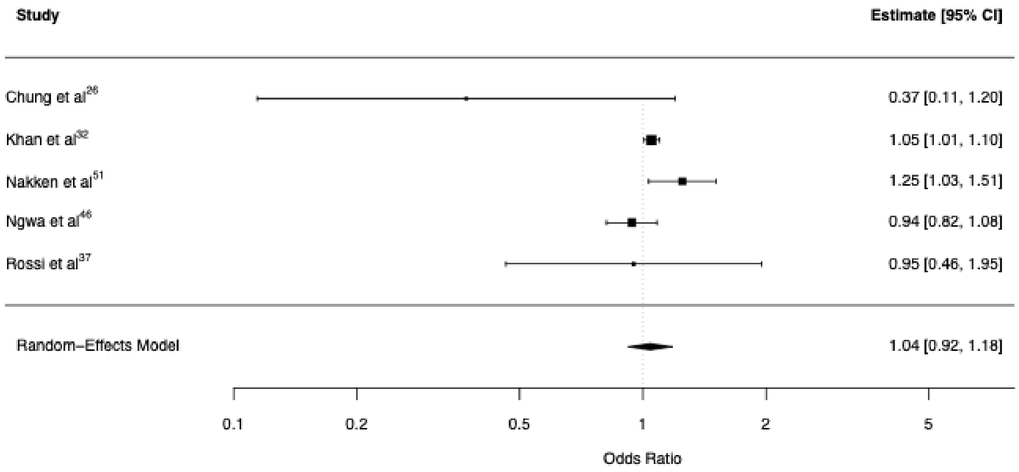


© 2025 Virk S et al. *JAMA Network Open*.

### iii. Number of children in family

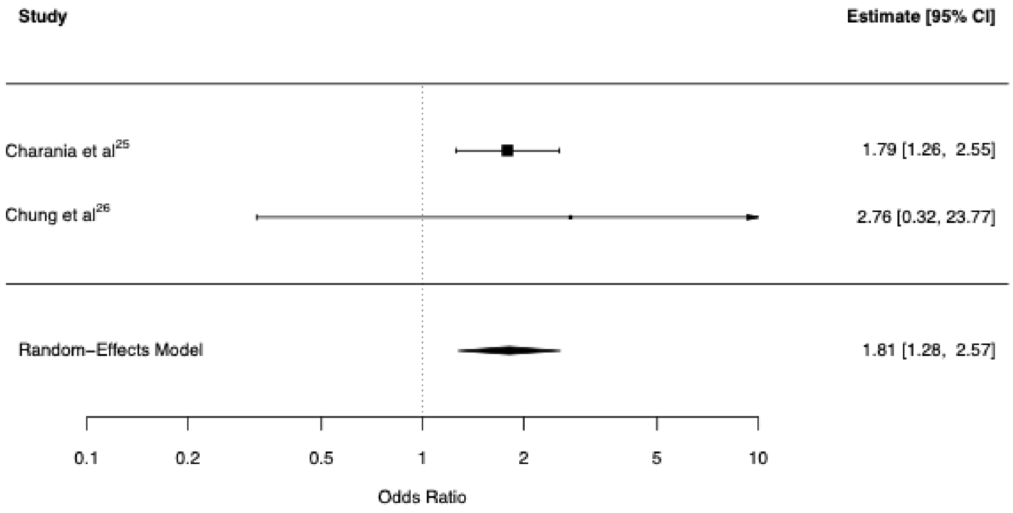


### iv. Guardian education

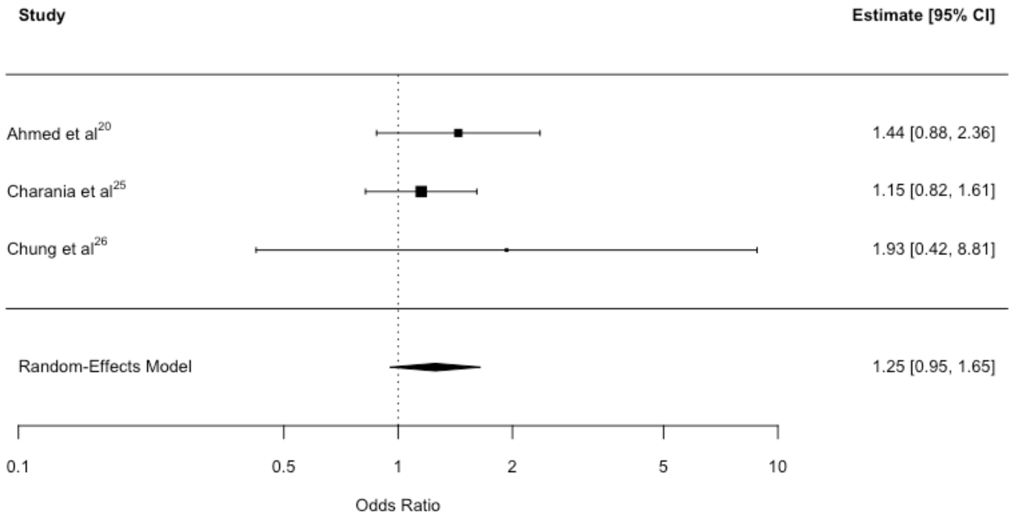


© 2025 Virk S et al. *JAMA Network Open*.

### v. Housing

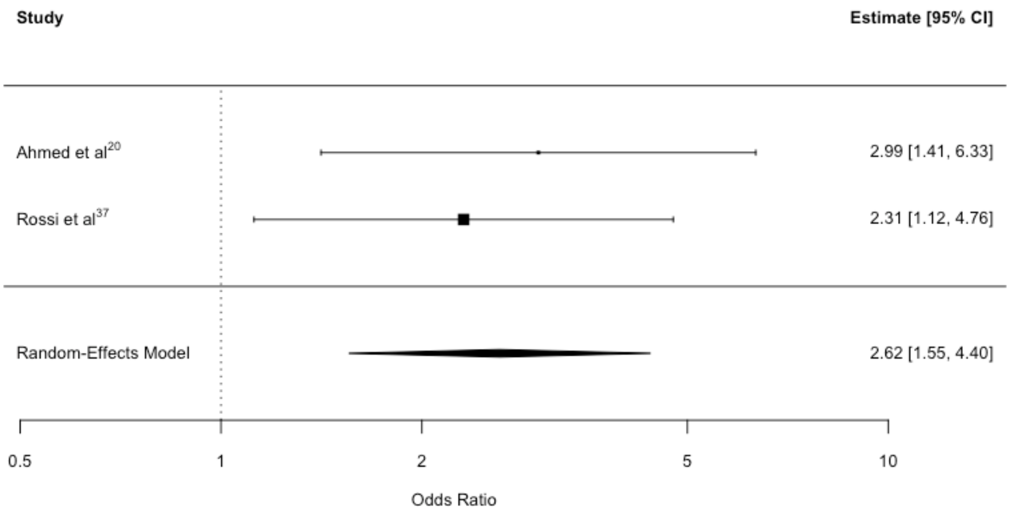


© 2025 Virk S et al. *JAMA Network Open*.

# eFigure 3. Influence Analysis for Vaccination Coverage

## A. Full Immunization

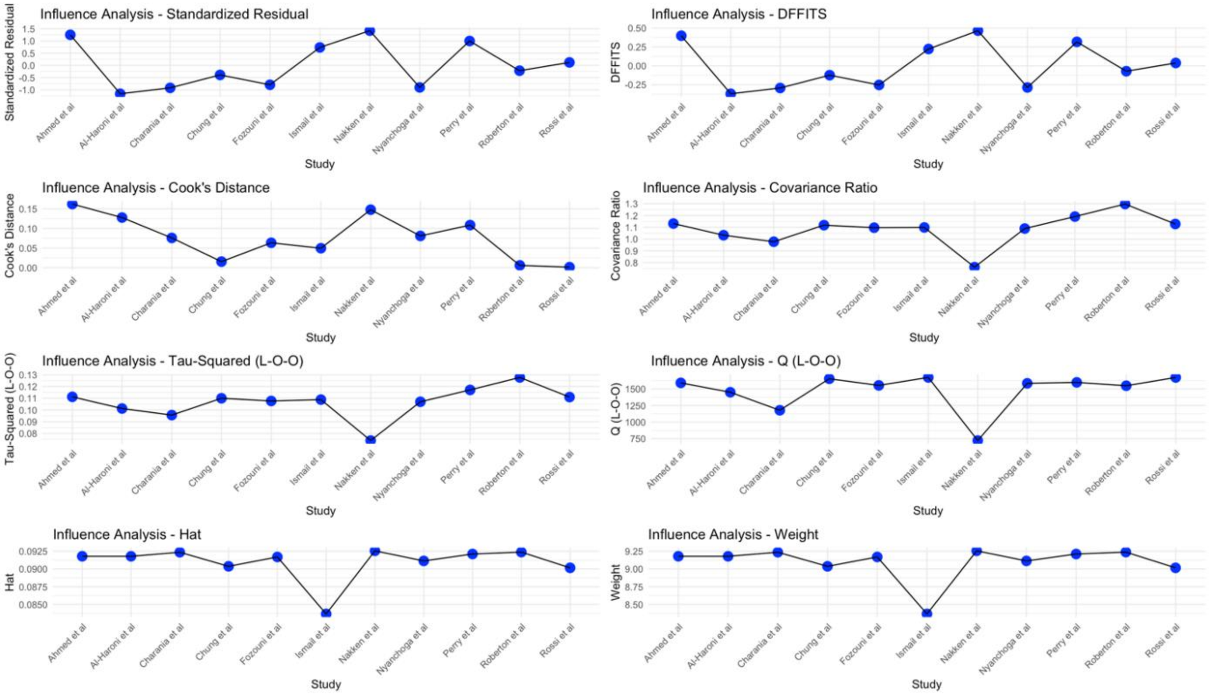


## B. Measles-Containing Vaccine

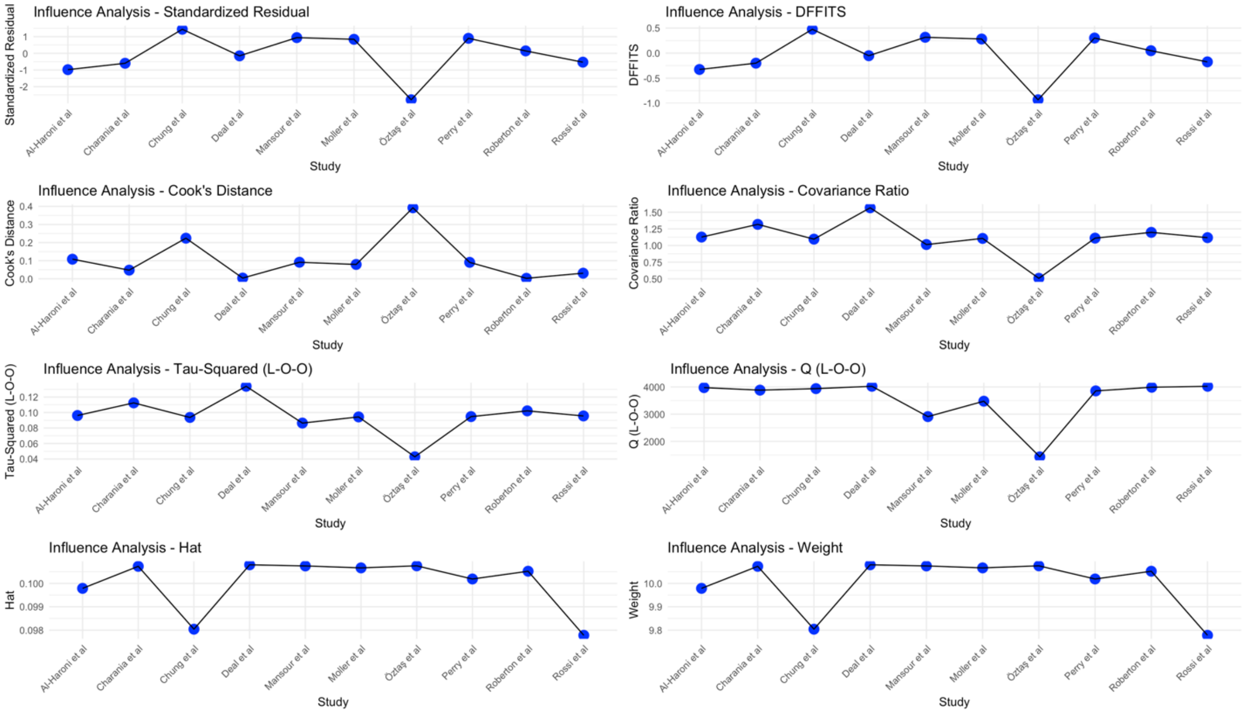


© 2025 Virk S et al. *JAMA Network Open*.

## C. Pertussis-Containing Vaccine

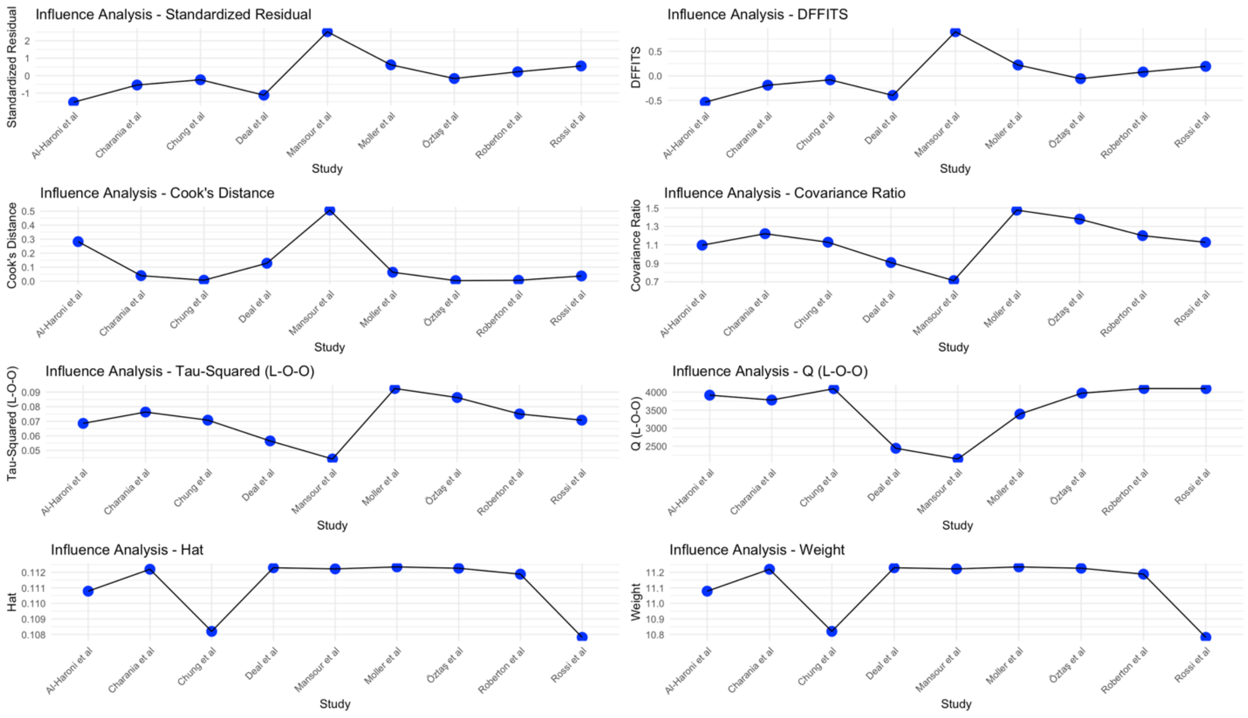


## D. Polio Vaccine

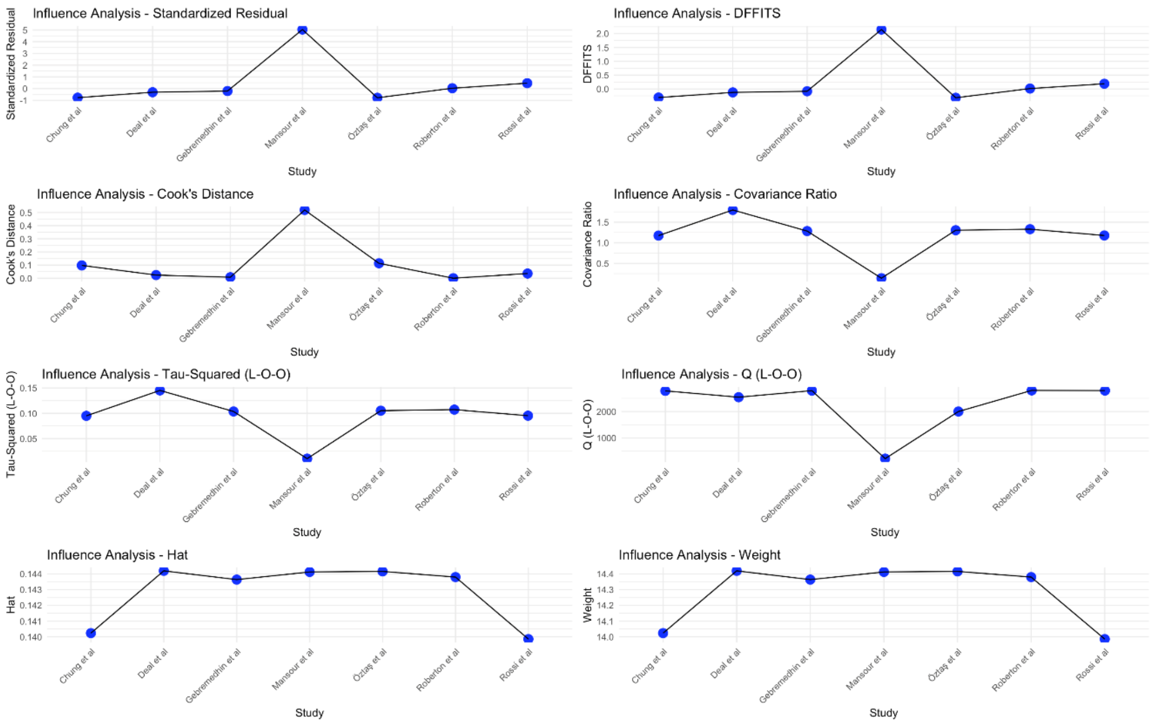


© 2025 Virk S et al. *JAMA Network Open*.

## E. Hepatitis B Vaccine

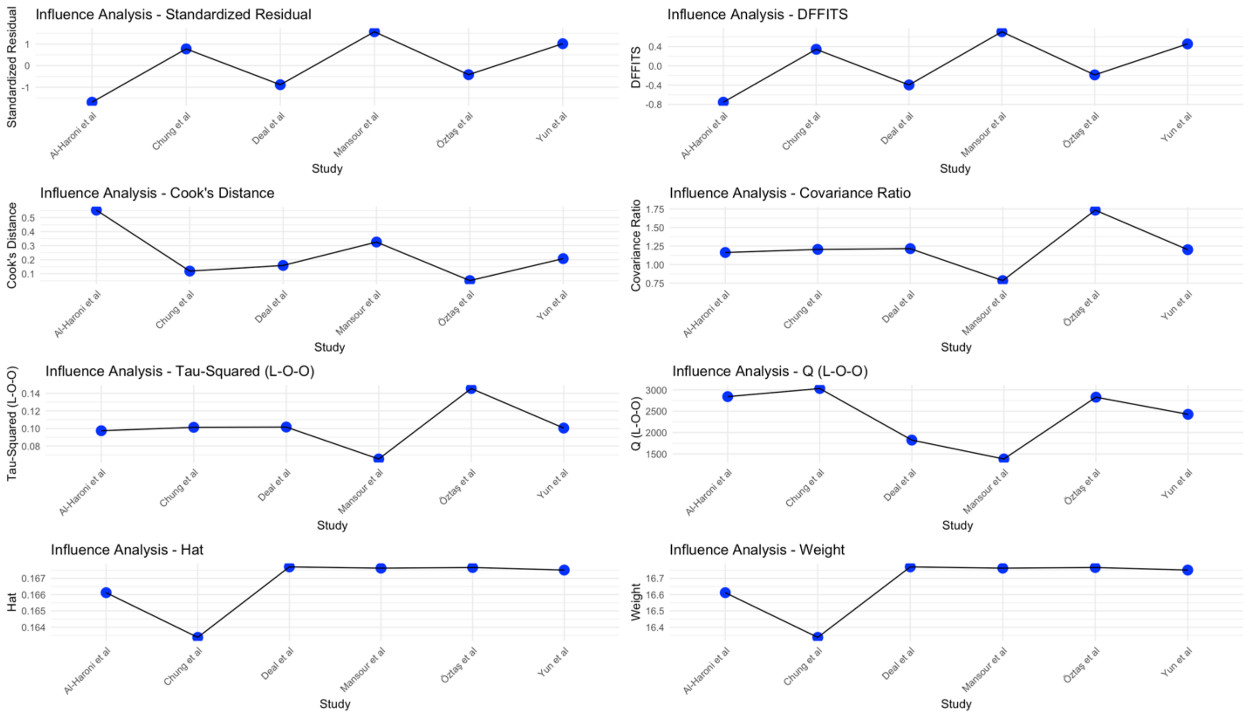


## F. BCG Vaccine

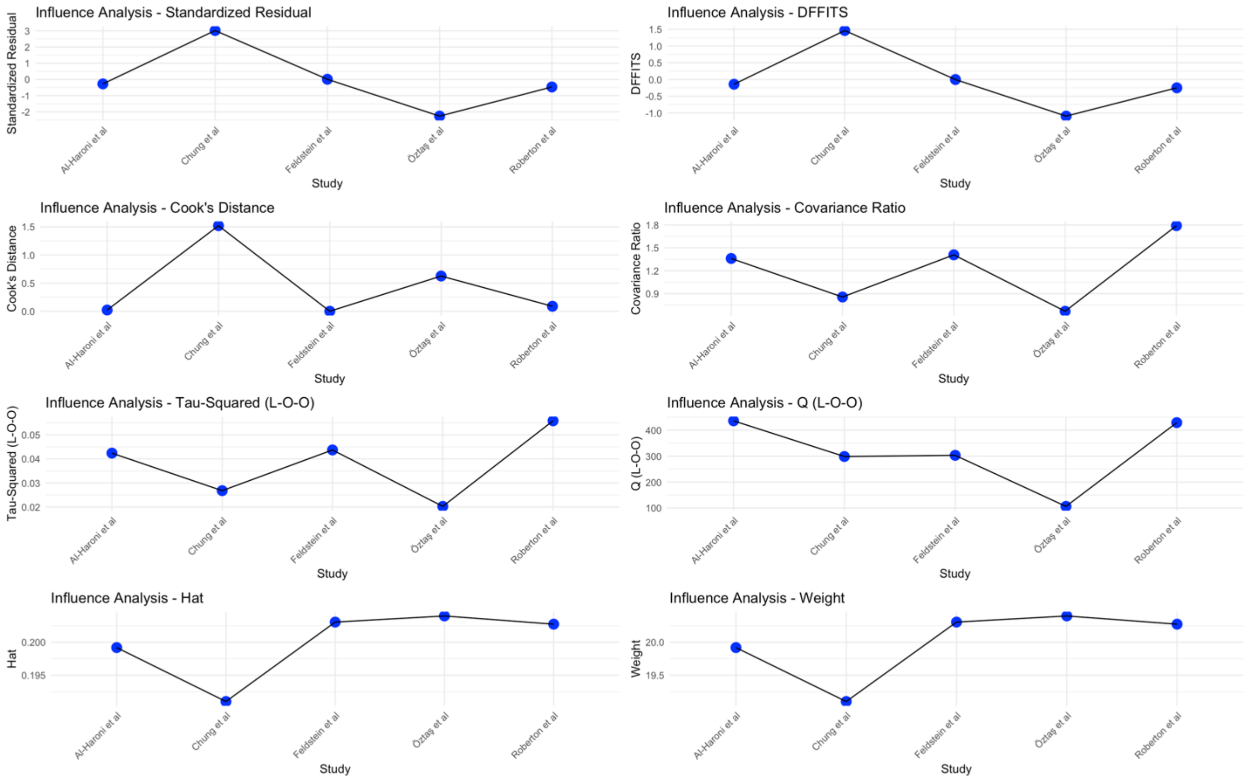

## eFigure 4: Publication Bias

**A. Full vaccination**

**i. Traditional funnel plot**

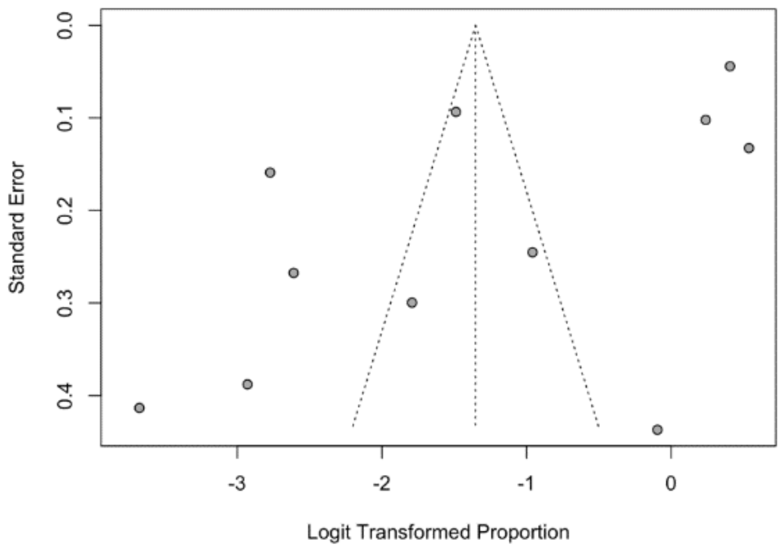


**ii. Alternative Funnel plot**

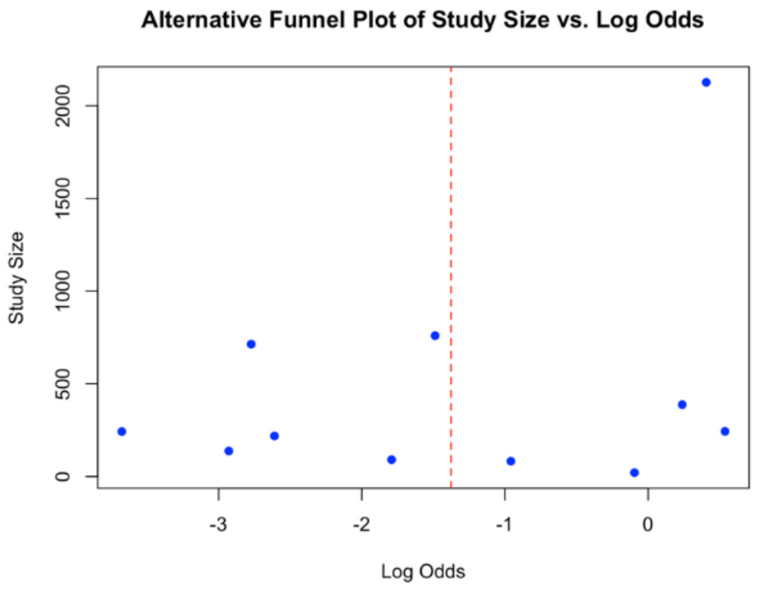


**iii. Begg's test**

Rank correlation test of funnel plot asymmetry :

Test result: z = -0.70, p-value = 0.4835

© 2025 Virk S et al. *JAMA Network Open*.

Bias estimate: -9.0000 (SE = 12.8452)

Reference: Begg & Mazumdar (1993), Biometrics

**B. Measles-Containing Vaccine**

**i. Traditional funnel plot**

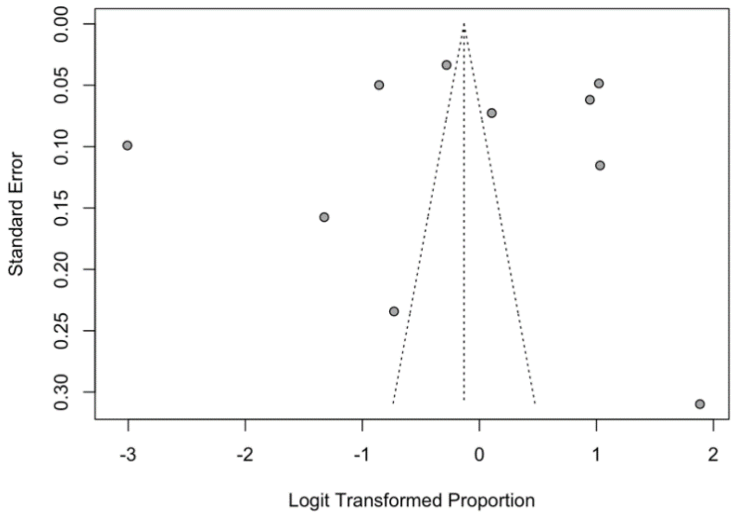


**ii. Alternative funnel plot**

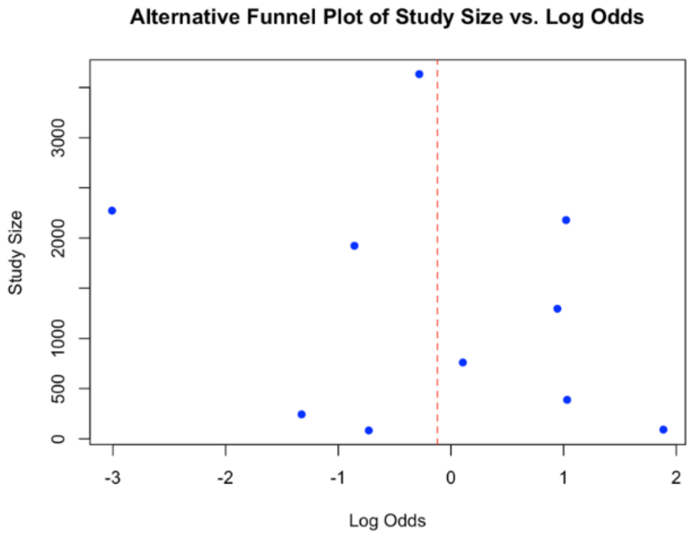


**iii. Begg's Test**

Rank correlation test of funnel plot asymmetry:

Test result: z = -0.27, p-value = 0.7884

© 2025 Virk S et al. *JAMA Network Open*.

Bias estimate: -3.0000 (SE = 11.1803)

Reference: Begg & Mazumdar (1993), Biometrics

© 2025 Virk S et al. *JAMA Network Open*.

## eFigure 5. Leave-One-Out Sensitivity Analysis for Factors With High Heterogeneity

A. Nationality

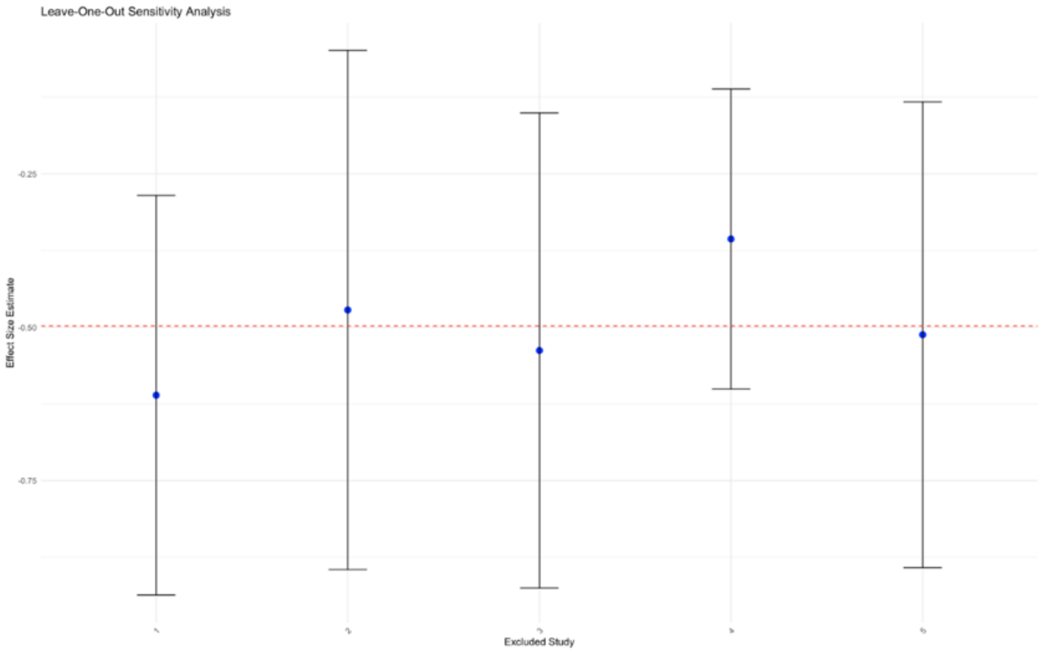


| | estimate | se | zval | pval | ci.lb | ci.ub | Q | Qp | tau2 | I2 | H2 |
|---|---|---|---|---|---|---|---|---|---|---|---|
| 1 | -0.6108 | 0.1662 | -3.6762 | 0.0002 | -0.9365 | -0.2852 | 28.5575 | 0.0000 | 0.0844 | 89.2776 | 9.3262 |
| 2 | -0.4719 | 0.2159 | -2.1860 | 0.0288 | -0.8950 | -0.0488 | 48.5981 | 0.0000 | 0.1541 | 89.4950 | 9.5193 |
| 3 | -0.5379 | 0.1975 | -2.7233 | 0.0065 | -0.9251 | -0.1508 | 49.0314 | 0.0000 | 0.1343 | 94.1955 | 17.2281 |
| 4 | -0.3562 | 0.1247 | -2.8558 | 0.0043 | -0.6007 | -0.1117 | 12.4359 | 0.0060 | 0.0380 | 69.4118 | 3.2692 |
| 5 | -0.5123 | 0.1937 | -2.6447 | 0.0082 | -0.8919 | -0.1326 | 51.2717 | 0.0000 | 0.1361 | 94.5300 | 18.2816 |

© 2025 Virk S et al. *JAMA Network Open*.

B. Household income

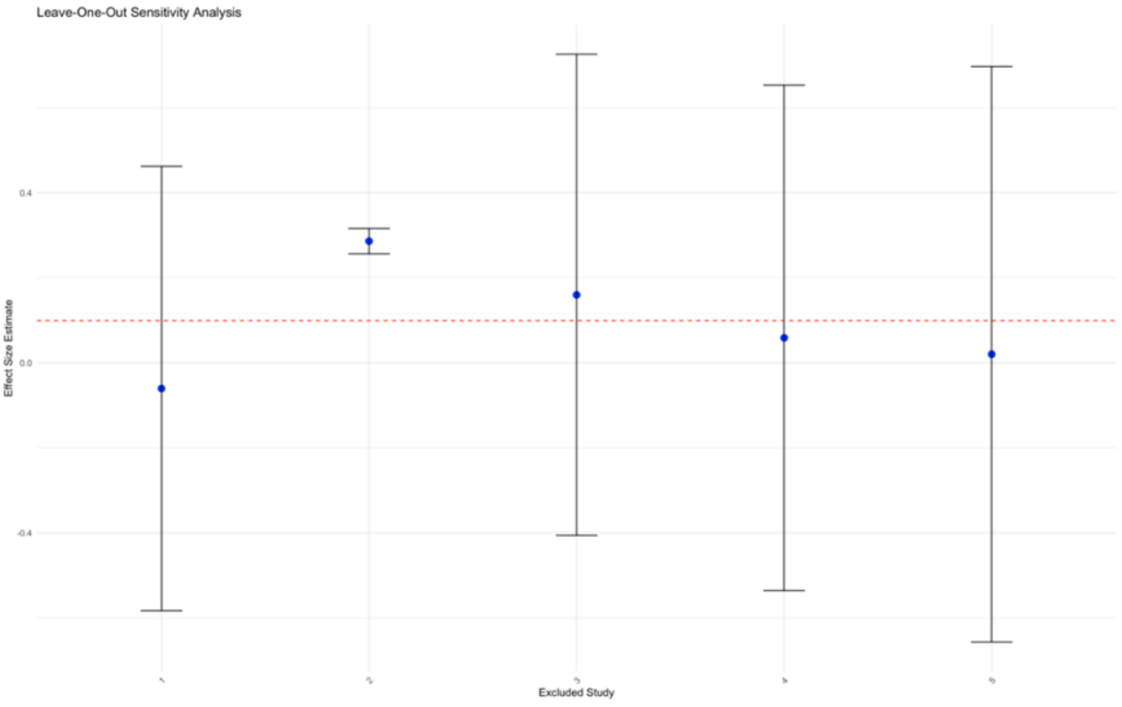


| | estimate | se | zval | pval | ci.lb | ci.ub | Q | Qp | tau2 | I2 | H2 |
|---|---|---|---|---|---|---|---|---|---|---|---|
| 1 | -0.0606 | 0.2665 | -0.2275 | 0.8200 | -0.5829 | 0.4617 | 12.6012 | 0.0056 | 0.1756 | 71.1080 | 3.4612 |
| 2 | 0.2858 | 0.0153 | 18.6623 | 0.0000 | 0.2558 | 0.3159 | 3.2458 | 0.3553 | 0.0000 | 0.0000 | 1.0000 |
| 3 | 0.1596 | 0.2886 | 0.5528 | 0.5804 | -0.4062 | 0.7253 | 13.4436 | 0.0038 | 0.2498 | 82.7494 | 5.7969 |
| 4 | 0.0584 | 0.3032 | 0.1928 | 0.8471 | -0.5358 | 0.6527 | 14.6525 | 0.0021 | 0.2780 | 83.9625 | 6.2354 |
| 5 | 0.0200 | 0.3452 | 0.0580 | 0.9537 | -0.6565 | 0.6966 | 11.6646 | 0.0086 | 0.3148 | 69.2262 | 3.2495 |

© 2025 Virk S et al. *JAMA Network Open*.

## eTable 5. Multiple Hypothesis Testing

| Unadjusted factors | p-value | Rank | BH critical value |
|---|---|---|---|
| father's employment | <0.0001 | 1 | 0.006 |
| household income | <0.0001 | 2 | 0.011 |
| housing | <0.0001 | 3 | 0.017 |
| area | <0.0001 | 4 | 0.022 |
| no. of children | 0.0018 | 5 | 0.028 |
| nationality | 0.0043 | 6 | 0.033 |
| guardian education | 0.0268 | 7 | 0.039 |
| age of guardian | 0.2379 | 8 | 0.044 |
| sex of child | 0.4387 | 9 | 0.05 |
| | | | |
| **Adjusted factor** | | | |
| Nationality | <0.0001 | 1 | 0.01 |
| Housing | 0.0003 | 2 | 0.02 |
| No.of children | 0.0009 | 3 | 0.03 |
| Guardian education | 0.1069 | 4 | 0.04 |
| Sex of child | 0.5192 | 5 | 0.05 |

© 2025 Virk S et al. *JAMA Network Open*.

## eTable 6. Quantitative Data for Reasons for Under-Vaccination

| | **Feldstein et al[30], 2020 (n=131)** | **Khan et al[32], 2019 (n= 18904)** | **Ngwa et al[46], 2020** | **Sheikh et al[39], 2014 (n=90)** |
|---|---|---|---|---|
| **Lack of awareness or information** | | | | |
| Not aware of campaign | 38.93129771 | 27.26 | 31.00 | 7.78 |
| Unaware of the place or date of the campaign (schedule) | | 12.79 | 18.5 | 52.23 |
| **Logistical issue** | | | | |
| Vaccination time inconvenient | | | | 4.45 |
| Vaccination site too far | | 0.35 | | 3.34 |
| Travelling | 3.816793893 | 14.21 | 35.5 | |
| Long queue | | 0.25 | | |
| vaccinator not present | | 3.95 | 10 | |
| no vaccine at the vaccination site | | 3.84 | | |
| **Health related concerns** | | | | |
| child sick | | 5.665467626 | | 5.56 |
| fear of vaccine/pain | 36.64122137 | 0.724714346 | | 3.34 |
| fear of adverse events | 3.053435115 | 0.232754972 | 5 | 2.23 |
| **Perception and attitude beliefs** | | | | |
| vaccine does not help | | | 4.5 | |
| discouraged by community | | | 13.5 | |
| did not give importance | 6.870229008 | 7.20 | | |
| child absent during vaccination activities | 34.35114504 | | | 6.67 |

© 2025 Virk S et al. *JAMA Network Open*.

**eTable 7. Barriers and Facilitator for Vaccination Campaign**

| Study | Barriers/Facilitators | Lessons from camps |
|---|---|---|
| **Dhoubhadel et al[42], 2017** | Barriers | Due to earthquake, transfer of vaccine delayed. No cold-chain facility in camp thus small number of vaccines had to be carried every day in cold-chain boxed to the field site from hospital. |
| | Facilitators | Local community support including local youth clubs, leaders and public health officials. |
| **Khan et al[32], 2019** | Barriers | Conducting campaign was challenging due to regular influx, scattered settlement in wide areas, population mobility within the camps, misconception on vaccination, and language barriers of the FDMN population. Short campaign duration, and the FDMNs' initial priority toward relief collection than vaccination were additional barriers. Relative complex administration of intramuscular vaccine as compared to oral. |
| | Facilitators | Multi-sectoral collaboration with WHO, UNICEF and other non-government organizations. |
| **Korave et al[45], 2021** | Barriers | IDP camps highly unstable (could account for low coverage). There is issue with denominator leading to high tendency to inflation. Challenge of funding was an additional barrier. |
| | Facilitators | Traditional and religious leaders essential for acceptance. (essential component of social mobilisation) |
| **Koop et al[33], 2001** | Barriers | The rapid evolution of the conflict and the significant turnover of the camps' populations created an environment in which children could and did miss opportunities to be vaccinated. |
| | Facilitators | Weekly clinics which provide immunisation. The clinics achieved rates above 90%, avoiding outbreak. |
| **Ngwa et al[46], 2020** | Barriers | Refer to Table 6 |
| | Facilitators | Protection from cholera; convinced by campaigners, CHW, local leaders; Followed others; recommended by doctors; free offer |
| **Rossi et al[37], 2016** | Barriers | Children living in collective shelters or tens were less vaccinated than those living in houses or apartments. |
| | Facilitators | Living in El-Bire which as a public primary care facility managed by Ministry of Social Affairs – MoSA . |
| **Sheikh et al[39], 2014** | Barriers | Vaccinator errors (mistake in injection technique and improper use of IPV) due to insufficient training. Cold chain issues with inconsistent temperature control, and inadequate planning. No vial monitors on IPV and staff unaware of freezing risks. |
| | Facilitators | 1) Strong commitment from the Ministry of Health and quick resource allocation, 2) flexibility to move "temporary fixed" sites frequently in response to caregiver demands to bring vaccine closer to their homes, and 3) High caregiver acceptance of IPV, with few refusals. |

© 2025 Virk S et al. *JAMA Network Open*.

**eTable 8. Down and Black Checklist Quality**

| Study Author | Scores | Category "Excellent" (score ≥ 26), "Good" (score 20-25), "Fair" (score 15-19), and "Poor" (score ≤ 14) |
|---|---|---|
| Ahmed et al[20], 2023 | 16 | Fair |
| Al-Haroni et al[21], 2023 | 16 | Fair |
| Charania et al[22], 2018 | 10 | Poor |
| Charania et al[23], 2023 | 18 | Fair |
| Charania et al[24],2023 | 18 | Fair |
| Charania et al[25], 2023 | 18 | Fair |
| Chauhan et al[50], 2019 | 16 | Fair |
| Chung et al[26], 2016 | 16 | Fair |
| Deal et al[27], 2022 | 15 | Fair |
| Debela et al[28], 2022 | 17 | Fair |
| Dhoubhadel et al[42], 2017 | 13 | Poor |
| El-Halabi et al[29], 2023 | 21 | Good |
| Feldstein et al[30], 2020 | 18 | Fair |
| Fozouni et al[31], 2019 | 19 | Fair |
| Gebremedhin et al[53], 2023 | 20 | Good |
| Ismail et al[43], 2014 | 17 | Fair |
| Kaewkungwal et al[44], 2010 | 21 | Good |
| Khan et al[32], 2019 | 17 | Fair |
| Klok-Nentjes et al[58], 2018 | 12 | Poor |
| Koop et al[33], 2001 | 10 | Poor |
| Korave et al[45], 2021 | 12 | Poor |
| Mansour et al[34], 2019 | 17 | Fair |
| Moller et al[54], 2016 | 17 | Fair |
| Moller et al[55], 2018 | 24 | Good |
| Nakken et al[51], 2018 | 17 | Fair |
| Ngwa et al[46], 2020 | 20 | Good |
| Nyanchoga et al[56], 2021 | 23 | Good |
| Oladeji et al[47], 2019 | 19 | Fair |
| Öztaş et al[35], 2020 | 19 | Fair |
| Perry et al[52], 2020 | 20 | Good |
| Roberton et al[36], 2016 | 19 | Fair |
| Rossi et al[37], 2016 | 21 | Good |
| Ruiz-Rodríguez et al[48], 2008 | 21 | Good |
| Seal et al[49], 2023 | 24 | Good |
| Sheikh et al[38], 2009 | 16 | Fair |
| Sheikh et al[39], 2014 | 14 | Poor |
| Shiferie et al[57], 2023 | 17 | Fair |
| Troiano et al[40], 2022 | 10 | Poor |
| Yun et al[41], 2016 | 24 | Good |

© 2025 Virk S et al. *JAMA Network Open*.

## eTable 9. GRADE Criteria: Level of Evidence for Each Outcome.

| Outcomes | Type of Evidence | Risk of bias | Inconsistency | Indirectness | Imprecision | Publication Bias | Large magnitude of effect | Dose Response | Effect of all plausible confounding factors | Overall Quality |
|---|---|---|---|---|---|---|---|---|---|---|
| **Vaccination coverage** | | | | | | | | | | |
| Full immunization | High quality | 0 | -1 | 0 | 0 | 0 | 0 | 0 | 0 | Medium |
| Vaccination campaign | High quality | -1 | -1 | 0 | 0 | 0 | 0 | 0 | 0 | Low |
| Measles Containing Vaccine | High quality | 0 | -1 | 0 | 0 | 0 | 0 | 0 | 0 | Medium |
| Pertussis Containing Vaccine | High quality | 0 | -1 | 0 | 0 | 0 | 0 | 0 | 0 | Medium |
| Polio | High quality | 0 | -1 | 0 | 0 | 0 | 0 | 0 | 0 | Medium |
| Hepatitis B | High quality | 0 | -1 | 0 | 0 | 0 | 0 | 0 | 0 | Medium |
| BCG | High quality | 0 | -1 | 0 | 0 | 0 | 0 | 0 | 0 | Medium |
| **Factors affecting vaccination** | | | | | | | | | | |
| Sex of child | Low quality | 0 | 0 | 0 | 0 | 0 | 0 | 0 | +1 | Medium |
| Father's occupation | Low quality | 0 | 0 | 0 | 0 | 0 | +1 | 0 | 0 | Medium |
| Nationality | Low quality | 0 | -1 | 0 | 0 | 0 | 0 | 0 | +1 | Low |
| Housing | Low quality | 0 | 0 | 0 | 0 | 0 | +1 | 0 | +1 | High |
| Guardian education | Low quality | 0 | -1 | 0 | 0 | 0 | 0 | 0 | +1 | Low |
| Number of children | Low quality | 0 | 0 | 0 | 0 | 0 | 0 | 0 | +1 | Medium |
| Household income | Low quality | 0 | -1 | 0 | 0 | 0 | 0 | 0 | 0 | Very low |

© 2025 Virk S et al. *JAMA Network Open*.

| | | | | | | | | | | |
|---|---|---|---|---|---|---|---|---|---|---|
| Age of Guardian | Low quality | 0 | 0 | 0 | 0 | 0 | 0 | 0 | 0 | Low |
| Area of residence | Low quality | 0 | 0 | 0 | 0 | 0 | +1 | 0 | 0 | Medium |

The overall score quality is dependent on the total sum of all the included categories.

**Vaccination coverage**

Observational studies start with a high quality of evidence in prevalence studies.

Risk of bias:

None of the studies included for coverage were high in the risk of bias, except the studies included for post-vaccination campaign since they were mostly reports. Thus, for post-campaign -1 has been done.

For inconsistency:

Although I2 is high, the variability can be explained with different study population characteristics, and different vaccine doses schedules, we were unable to analyze subgroup differences.

For indirectness:

Not at all. Study population is representative of the population under study.

For imprecision:

CI are not too wide, subjective.

Publication bias:

Begg's test showed no publication bias for vaccination coverage and MCV coverage. (only coverage with more than 10 studies)

Large magnitude of effect:

No.

Dose Response relationship:

None.

Effect of all plausible confounding factors:

Not used for proportion systematic reviews due to bias.

**Factors affecting vaccination**

For risk of bias:

If you have conducted a meta-analysis, consider conducting a sensitivity analysis with only low risk of bias studies. If the effect estimates are unchanged then you may be confident that the risk of bias of the studies does not alter the results

For inconsistency:

There is overlap with CIs associated with effect estimates. Although I2 is high in some factors, the variability can be explained with different study population characteristics. Some inconsistency still might exist –1.

For indirectness:

© 2025 Virk S et al. *JAMA Network Open*.

Not at all (does not appear to be an issue).

For imprecision:

For dichotomous outcome, event rates at least need to be 300.

Publication bias:

Funnel plots were analyzed and on visualization no asymmetry was evident.

Large magnitude of effect:

Studies with OR>2 or <0.5 (based on consistent evidence from at least 2 studies).

Dose Response relationship:

Not Applicable

Effect of all plausible confounding factors:

Factors in which adjusted odds ratio could be analyzed have been given +1.

© 2025 Virk S et al. *JAMA Network Open*.

**eTable 10: Factors That Need Further Exploration**

| |
|---|
| Age of child |
| Visa category |
| Arrival year |
| Region of origin |
| Ethnicity |
| Religion |
| Sex of caregiver |
| Family size |
| Parent language spoken |
| Electronic media |
| Children lost |
| Child ever admitted to hospital |
| Immunization session attended |
| Vaccine knowledge |
| Vaccine attitude/preference |
| Marital status of guardian |
| Fear of arrest |
| Cost of vaccination |
| Access of health services |
| Child's country of birth |
| Child's place of birth |
| Vaccination card |
| Previous miscarriage |
| First-Born status |
| Duration of stay |
| Decision maker in family |
| Health insurance status |
| Vaccine team respect |
| Utilization of clinic |

© 2025 Virk S et al. *JAMA Network Open*.
