# Supplementary material for: Vaccination in Forcibly Displaced, Pediatric Populations: A Systematic Review and Meta-Analysis
Source: JAMA Netw Open. 2025 Jun 16;8(6):e2516237. doi: 10.1001/jamanetworkopen.2025.16237 (PMC12171943; doi:10.1001/jamanetworkopen.2025.16237)
Supplement: Supplement 2. — Data Sharing Statement [file jamanetwopen-e2516237-s002.pdf]

Data Sharing Statement

Virk. Vaccination in Forcibly Displaced, Pediatric Populations. *JAMA Netw Open*. Published June 16, 2025. doi:10.1001/jamanetworkopen.2025.16237

Data
Data available: Yes
Data types: Data (not involving human participants)
How to access data: contact: sav4017@med.cornell.edu
When available: With publication

Supporting Documents
Document types: Statistical/analytic code
How to access documents: sav4017@med.cornell.edu
When available: With publication

Additional Information
Who can access the data: anyone requesting
Types of analyses: for any purpose
Mechanisms of data availability: with investigator support
Any additional restrictions: NA
